# The histone replacement gene *His4r* is involved in heat stress induced chromatin rearrangement

**DOI:** 10.1038/s41598-021-84413-4

**Published:** 2021-03-01

**Authors:** Anikó Faragó, Adél Ürmösi, Anita Farkas, László Bodai

**Affiliations:** 1 Department of Biochemistry and Molecular Biology, Faculty of Science and Informatics, University of Szeged, Közép fasor 52, 6726 Szeged, Hungary; 2 Doctoral School in Biology, Faculty of Science and Informatics, University of Szeged, 6726 Szeged, Hungary

**Keywords:** Chromatin structure, Histone variants, Circadian rhythms, Chromatin analysis, Drosophila, Gene expression analysis, Reverse transcription polymerase chain reaction, Genetic techniques, PCR-based techniques, Gene expression profiling, Genome-wide analysis of gene expression, DNA sequencing, Next-generation sequencing, Ageing, Epigenetics, Eukaryote, Functional genomics, Gene expression, Gene regulation, Mutation, Chromatin, Epigenetics, Transcription, Transcriptomics

## Abstract

His4r is the only known variant of histone H4 in *Drosophila*. It is encoded by the *His4r* single-copy gene that is located outside of the histone gene cluster and expressed in a different pattern than H4, although the encoded polypeptides are identical. We generated a null mutant (*His4r*^*Δ42*^) which is homozygous viable and fertile without any apparent morphological defects. Heterozygous *His4r*^*Δ42*^ is a mild suppressor of position-effect variegation, suggesting that His4r has a role in the formation or maintenance of condensed chromatin. Under standard conditions loss of His4r has a modest effect on gene expression. Upon heat-stress the induction of the Heat shock protein (HSP) genes *Hsp27* and *Hsp68* is stronger in *His4r*^*Δ42*^ mutants with concordantly increased survival rate. Analysis of chromatin accessibility after heat shock at a *Hsp27* regulatory region showed less condensed chromatin in the absence of His4r while there was no difference at the gene body. Interestingly, preconditioning before heat shock led to increased chromatin accessibility, HSP gene transcription and survival rate in control flies while it did not cause notable changes in *His4r*^*Δ42*^. Thus, our results suggest that His4r might play a role in fine tuning chromatin structure at inducible gene promoters upon environmental stress conditions.

## Introduction

Packaging of chromosomal DNA in the nucleus is accomplished by the formation of chromatin structure which is a complex of DNA, RNA, and histone and non-histone proteins^1,2^. The basic repeating unit of chromatin is the nucleosome which consists of 147 bp DNA wrapped around an octamer of core histones H2A, H2B, H3 and H4^1,3^. The chromatin fiber can go through further condensation and form either loosely packed euchromatin or more condensed heterochromatin^4^. The chromatin structure hinders the accessibility of DNA to non-histone proteins that control replication, gene expression or DNA repair. In favor of these events chromatin is relaxed in a coordinated and organized way through chromatin remodeling or histone modifications^5^.

One way of chromatin remodeling is the replacement of nucleosomal histones by histone variants^6^. In higher eukaryotes replication dependent, so called canonical histone genes can be found in tandem copies in histone gene clusters that are mainly active during the S phase of the cell cycle. Genes of canonical histones do not possess introns and their transcripts do not have poly(A) tails. Histone variants, on the other hand, are encoded by intron containing single-copy genes that express polyadenilated mRNA in nondividing, terminally differentiated tissues^7,8^. All known histone variant genes encode proteins that differ from the canonical histones^9^, except for one, histone H4.

In *Drosophila* a single-copy histone H4 variant gene was identified by Akhmanova et al. that encodes a protein with the same amino acid sequence as canonical H4 and was consequently named *H4 replacement* (CG3379, henceforth *His4r*) as due to their identity at the protein level His4r is a replacement histone not a variant^10^. Unlike canonical histone *H4* genes *His4r* contains 2 introns and its transcript is polyadenilated^10,11^ and similarly to other histone variant genes^12,13^ it is localized outside of the histone gene cluster. According to high-throughput expression data *His4r* has a relatively uniform expression profile in all tissues and developmental stages^14,15^ suggesting that it serves functions utilized by all cell types and that its specific functions might be defined by post-transcriptional processes^11^.

Among closely related *Drosophila* species the gene structure of *His4r* is very similar with only minor differences in the nucleotide sequences encoding for identical proteins. This strong conservation led to the assumption that His4r, similarly to H3.3, might play a role in histone replacement^11^.

To date there are only a few reports about H4 replacement histones in other species like H4V in *Trypanosoma*^16^, H4v in *Neurospora crassa*^17^ or bovine H4-v.1^18^. Although H4 replacement genes have been identified in several species no functional characterization has been reported and their functions are yet to be clarified.

To analyze *Drosophila melanogaster His4r* we generated a null mutant (*His4r*^*Δ42*^) by deleting the entire coding region of the *His4r* gene and performed phenotypic characterization assays. *His4r*^*Δ42*^ flies are homozygous viable and fertile, however, we observed mild sublethality in females. We performed position-effect variegation (PEV) and transcriptome analysis as His4r being a histone protein is expected to have a role in chromatin organization and found that loss of *His4r* acts as a mild PEV suppressor and has a modest effect on gene expression. To analyze the role of His4r in an inducible gene expression setting we investigated transcriptional and chromatin accessibility changes of Heat shock protein (HSP) genes upon heat shock treatment and found that lack of His4r has effects similar to preconditioning: increased HSP transcription, increased chromatin accessibility of the *Hsp27* promoter, and improved survival. Thus, our results indicate that His4r plays a role in the formation of local chromatin structure at inducible gene promoters.

## Results

### Generation of the *His4r*^*∆42*^ null allele

**Figure 1:**
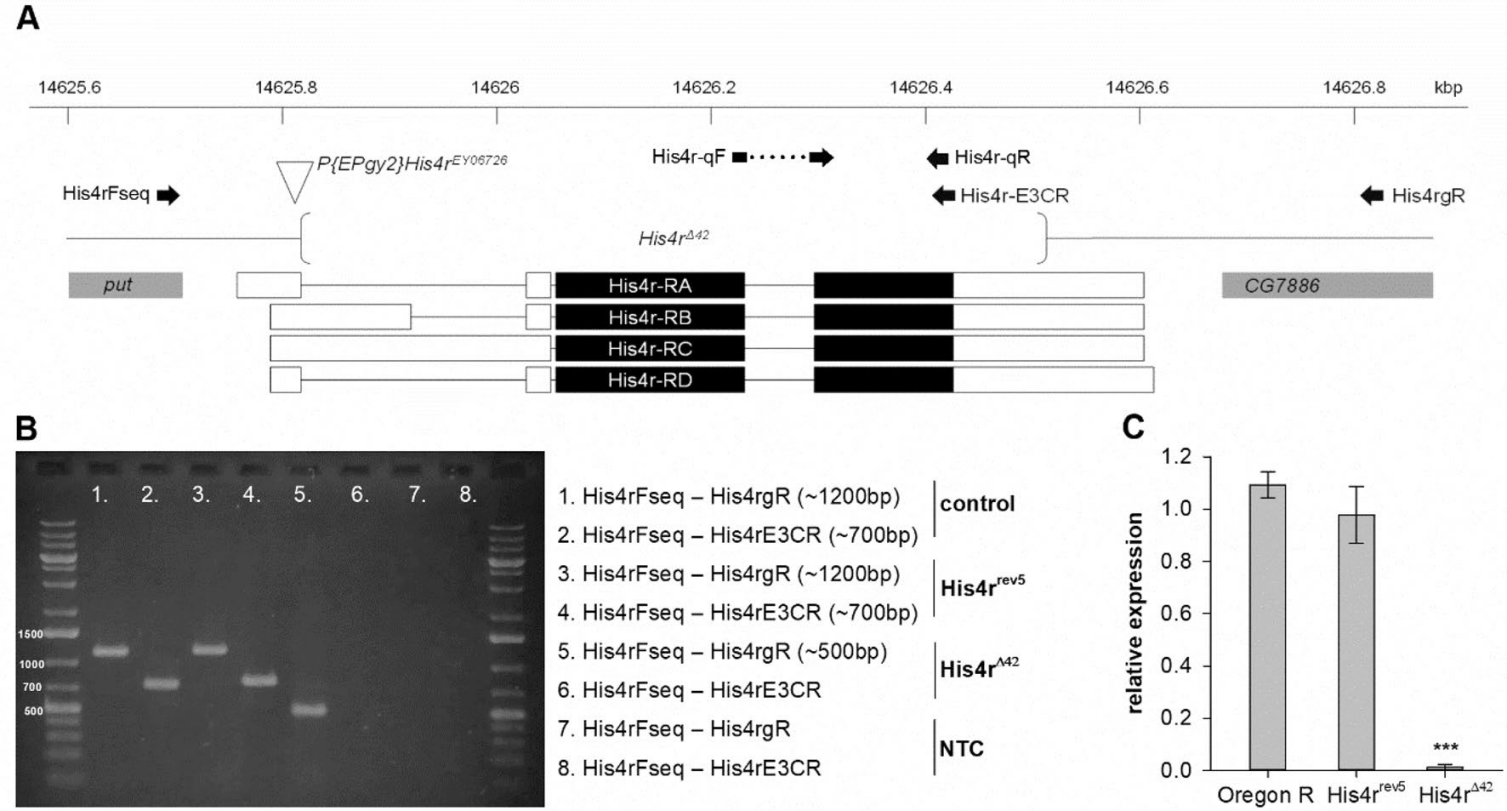
Generation and validation of the *His4r*^*Δ42*^ deletion mutant. **(A)** The genomic region of *His4r* on chromosome arm 3R with transcript models, primers and the extent of the deletion is shown. On the *His4r* transcript models coding sequences are represented by black bars, untranslated exonic regions by empty bars, and introns by solid lines. Adjacent ends of neighboring genes (*put* and *CG7886*) are shown with grey bars. Empty arrowhead indicates the position of the *P{EPgy2}His4r*^*EY06726*^ transposon in the *His4r* 5′-UTR, brackets show the breakpoints of the *His4r*^*Δ42*^ deletion. Position of PCR primers are marked by black arrows. **(B)** PCR reactions on homozygous *His4r*^*Δ42*^ template with His4rFseq and His4r-E3CR primers did not give a PCR product, with His4rFseq and His4rgR primers the length of the product was approximately 700 bp shorter than on wild-type control template. PCR reactions on *His4r*^*rev5*^ templates resulted in amplicons similar to wild-type. **(C)** *His4r* transcript levels were similar in *Oregon-R* (wild-type) and in *His4r*^*rev5*^ revertant flies (P > 0.05), while in *His4r*^*Δ42*^ mutants *His4r* mRNA was not present (P = 2.69077 × 10^−5^, t-test). The bars show the mean of normalized RT-qPCR measurements relative to a template dilution series, error bars represent standard error, n = 3.

To be able to investigate in vivo functions of the *Histone H4 replacement* (*His4r*, CG3379) gene we generated null alleles by remobilization of the transposon inserted in the 5′-UTR of *His4r* at genomic position 3R:14,625,809 (Dm r6.15, FB2017_06) in the homozygous viable *P{EPgy2}His4r*^*EY06726*^ line. After remobilization of the P{EPgy2} element we selected revertants based on loss of the dominant *mini-white* marker gene and identified mutants in which deletions were generated as a consequence of imprecise transposon removal by screening revertants with PCR using primers straddling the *His4r* gene (Fig. 1A). The His4rFseq and His4rgR primers used are located -55 bp upstream and + 211 bp downstream of the *His4r* locus [3R:14,625,755.0.14,626,614], respectively, and produce a 1162 bp PCR product on wild-type template. Among over 200 tested revertants we found a single candidate, *His4r*^*Δ42*^, which gave a shorter, approximately 500 bp long PCR amplicon (Fig. 1B, Supplementary Fig. S1). PCR reaction on homozygous *His4r*^*Δ42*^ template with His4rFseq and His4rE3CR primers, the latter of which is located at the 3′ end of *His4r* coding sequence, did not produce a PCR amplicon suggesting that the deletion removes the coding region of *His4r* (Fig. 1B). We verified these results and determined the extent of the deletion by capillary sequencing. In *His4r*^*Δ42*^ a 694 bp deletion extends from a breakpoint in the 5′-UTR (3R:14,625,816) of *His4r* to one in the 3′-UTR (3R:14,626,511) removing the entire coding region but not affecting neighboring genes (Fig. 1A). The breakpoints enclose a 29 bp scar sequence containing remnants of the *P{EPgy2}* element (Supplementary Data S1).

We also selected and characterized a revertant line, *His4r*^*rev5*^, to use as a control in later experiments. By PCR genotyping (Fig. 1B) and capillary sequencing we found that in this line the remobilized P{EPgy2} transposon did not cause alterations in the gene sequence except for a 40 bp transposon derived scar sequence in the 5′-UTR straddled by 8 bp target site duplication (3R:14,625,809–14,625,816) (Supplementary Data S1). To determine whether the residual scar sequence affects the expression of *His4r* we compared *His4r* transcript levels in *Oregon-R* (wild-type), *His4r*^*rev5*^ and *His4r*^*Δ42*^ flies by reverse transcription quantitative PCR (RT-qPCR) and found that while *His4r* transcript was detected only at noise level in the deletion mutant its expression was not affected in the revertant strain (Fig. 1C).

### Loss of *His4r* causes reduced viability but does not affect fertility

**Figure 2:**
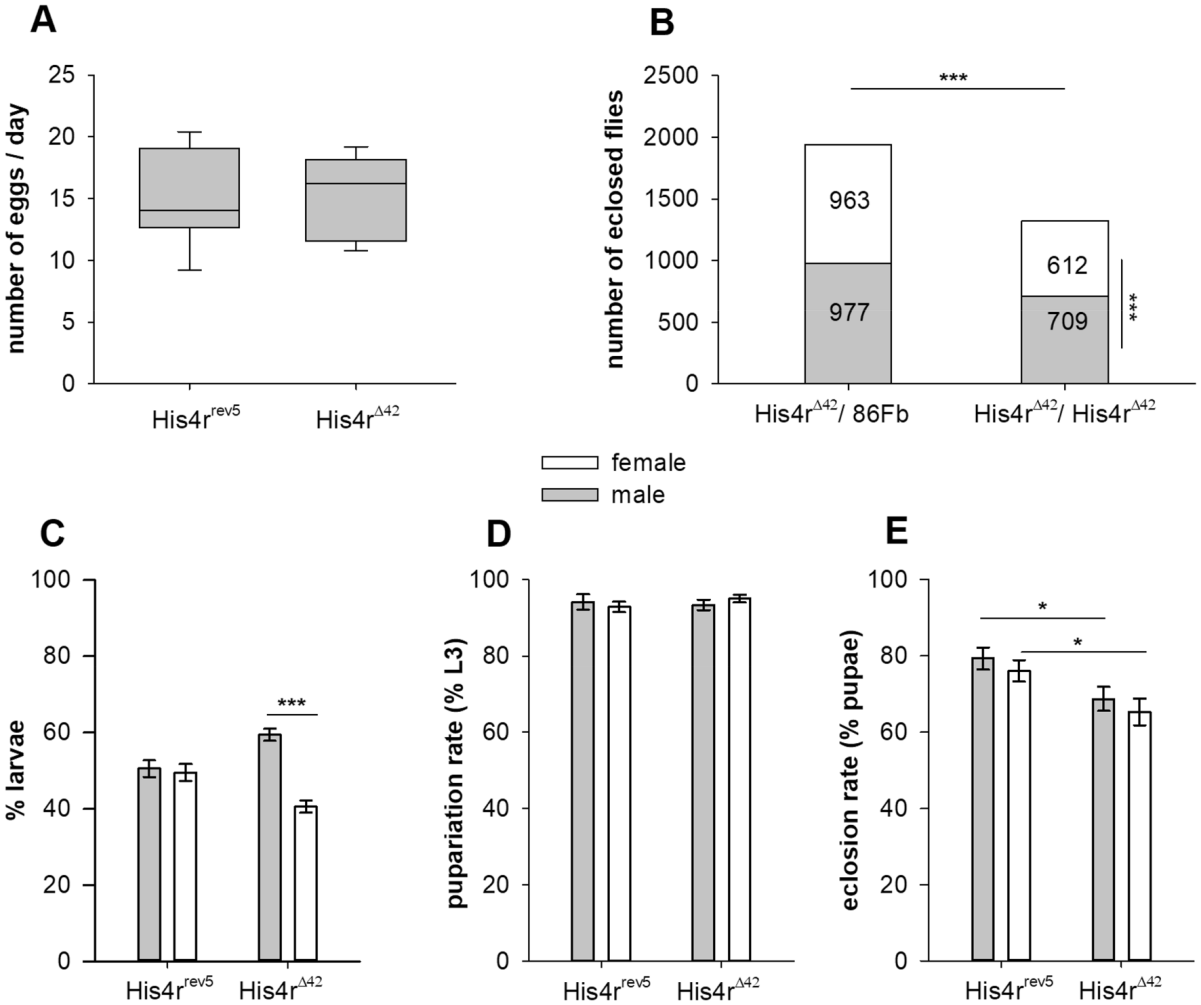
Loss of *His4r* reduces viability but does not affect fertility. **(A)** There was no significant difference in the fecundity of *His4r*^*Δ42*^ and *His4r*^*rev5*^ control females. Boxplot shows the distribution (first quartile, median, third quartile, and 10th and 90th percentiles as whiskers) of eggs laid by females daily, n = 5 females in 10 vials per each genotype. **(B)** The number of eclosed homozygous *His4r*^*Δ42*^ flies was significantly lower (P < 10^−6^, binomial test) than that of heterozygous control siblings and among homozygous *His4r*^*Δ42*^ progeny the number of females was significantly lower than that of males (P = 6.2 × 10^−4^, binomial test). **(C)** Among *His4r*^*Δ42*^ homozygous L3 larvae the ratio of females to males was significantly lower (P = 3 × 10^−10^, t-test) than among *His4r*^*rev5*^ controls. The bars show the percentage of male and female L3 larvae, error bars represent standard error, n = 20 vials per genotype. **(D)** The pupariation rate of *His4r*^*Δ42*^ homozygotes did not change compared to *His4r*^*rev5*^ controls. The bars show the percentage of pupariated L3 larvae, error bars represent standard error. **(E)** In *His4r*^*Δ42*^ homozygotes the eclosion rate of both male and female pupae were lower (P = 0.01 and P = 0.02, respectively, t-test) than that of the *His4r*^*rev5*^ controls. The bars show the percentage of eclosed pupae, error bars represent standard error. *P ≤ 0.05, ***P < 10^−3^.

Similarly to the *P{EPgy2}His4r*^*EY06726*^ parent strain the generated *His4r*^*Δ42*^ strain proved to be homozygous viable and fertile without any apparent morphological defects. To determine whether loss of *His4r* causes any phenotypic abnormalities or His4r functions are redundant we performed tests to assess potential changes in fertility, viability and longevity. Previously it was reported that flies homozygous for the piggyBac induced insertion allele *His4r*^*LL05512*^^19^ were sterile^20^. To clear this contradiction we performed complementation tests. We crossed male or female *w; His4r*^*LL05512*^*/His4r *^*Δ42*^ heterozygotes with *w*^*1118*^ flies and found that both crosses resulted in viable progeny (Supplementary Fig. S2). Thus, we conclude that the sterility of the *His4r*^*LL05512*^ strain is probably caused by a background mutation. Next, we performed fecundity assays and found that the egg laying capacity of homozygous *His4r*^*Δ42*^ females (15.3 ± 0.99 eggs/female/day) was similar to that of *His4r*^*rev5*^ control females (15.04 ± 1.2 eggs/female/day) (Fig. 2A). To test the viability of *His4r*^*Δ42*^ mutants we set up crosses to compare the eclosion rates of *His4r*^*Δ42*^ homozygous and heterozygous siblings. The number of homozygous *His4r*^*Δ42*^ offspring emerging from these crosses were lower than the number of heterozygous siblings (1321 vs 1940, respectively, P < 10^−6^, binomial test) (Fig. 2B), and the percentage of homo- and heterozygous offspring per vial (40.6 ± 0.6% and 59.4 ± 0.6%, respectively) significantly deviated from the expected 1:1 ratio (P = 8 × 10^–32^, t-test). These findings suggest that the deletion of *His4r* causes a sublethal developmental defect. We also observed that among the homozygous *His4r*^*Δ42*^ progeny the number of females was significantly lower than that of males (612 vs 709, respectively, P = 6.2 × 10^–4^, binomial test) (Fig. 2B). To determine whether females are lost before or after pupariation we compared the male:female ratio of *His4r*^*Δ42*^ mutants and *His4r*^*rev5*^ controls at different developmental stages. During the wandering L3 larval stage the average ratio of males and females in the *His4r*^*rev5*^ control strain was very close to the expected 50–50% while in the case of *His4r*^*Δ42*^ it shifted to 59.4 ± 1.6% males and 40.6 ± 1.6% females (P = 3 × 10^–10^, t-test) (Fig. 2C). When examining the pupation rates of the *His4r*^*Δ42*^ male or female larvae no significant loss could be observed compared to the controls (Fig. 2D). The eclosion rates of both *His4r*^*Δ42*^ male and female pupae were lower (P = 0.01 and P = 0.02, respectively, t-test) than that of the controls, however, there was no significant difference between the eclosion rates of the two genders (Fig. 2E). In conclusion, we could observe significant loss of *His4r*^*Δ42*^ individuals both during pupal development and during the development stages before pupation. The distorted male:female ratio of *His4r*^*Δ42*^ flies is resulted from the loss of females during embryonic or larval development but is not exacerbated during metamorphosis.

### The expression of *His4r* decreases during aging but its loss does not affect lifespan

**Figure 3:**
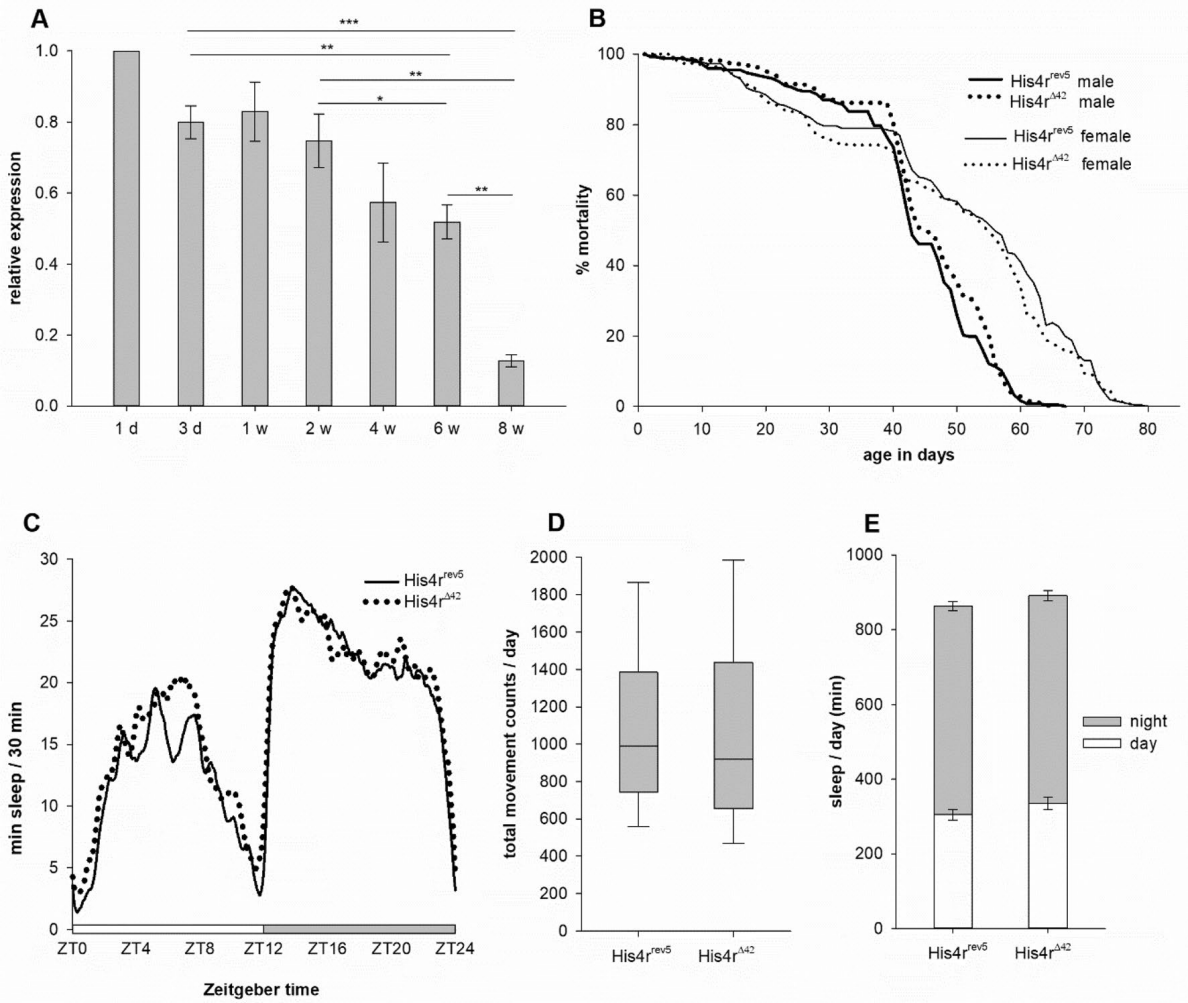
*His4r* is down-regulated with age but its loss does not affect longevity or circadian activity. **(A)** During the first 8 weeks of adult life *His4r* expression gradually decreased in male heads. Bars show the mean of normalized RT-qPCR measurements relative to a template dilution series, error bars represent standard error (n ≥ 4). *P < 0.05, **P < 10^−2^, ***P < 10^−3^ (OWA with Tukey HSD post-hoc test). **(B)** The longevity of homozygous *His4r*^*Δ42*^ males and females was similar to that of their *His4r*^*rev5*^ control counterparts. The graph shows the percent of survivors as function of age (n ≥ 240 in each category). **(C)** No notable difference could be observed between the sleep patterns of homozygous *His4r*^*Δ42*^ (n = 93) and *His4r*^*rev5*^ (n = 95) control males. **(D)** The daily movement of homozygous *His4r*^*Δ42*^ and *His4r*^*rev5*^ control males was also similar. Boxplot shows the distribution (first quartile, median, third quartile, and 10th and 90th percentiles as whiskers) of movement counts of individual flies per day. **(E)** The total amount of time that homozygous *His4r*^*Δ42*^ and *His4r*^*rev5*^ control males spent asleep was also similar both daytime and nighttime.

As besides mild sublethality we did not detect substantial developmental defects in *His4r*^*Δ42*^ flies, we turned our attention to adult phenotypes. First, we characterized *His4r* gene expression during aging in wild–type (*w*^*1118*^) flies by measuring transcript levels at set time points using RT-qPCR. During the first 8 weeks of adult life we observed a gradual decrease in the expression of *His4r* which can be considered a silencing of gene activity with age (P = 6.1004 × 10^–8^, One-Way ANOVA). During the first week the expression of *His4r* stabilized at around 80% of the level measured at the first day after eclosion from the pupal case, by weeks 4–6 it dropped to 50–60% and by the end of the second month it was as low as 12% of the initial level (Fig. 3A). Based on these expression data *His4r* transcription declines during the normal aging process. However, when performing longevity assays we did not detect major differences either in the time of 50% mortality rate (males: *His4r*^*rev5*^ 42.83 days vs. *His4r*^*Δ42*^ 44.63 days; females: *His4r*^*rev5*^ 54.9 days vs. *His4r*^*Δ42*^ 54.75 days) or in maximum lifespan (males: *His4r*^*rev5*^ 67 days vs. *His4r*^*Δ42*^ 65 days; females: *His4r*^*rev5*^ 79 days vs. *His4r*^*Δ42*^ 78 days) between the *His4r* mutant and the control strain (Fig. 3B).

### Loss of *His4r* does not affect circadian activity

Recent studies revealed that transcription-permissive chromatin states are dynamically established in a circadian-time-specific manner, thus, chromatin remodeling appears to be a major regulatory mechanism in the circadian machinery^21–23^. Since His4r has been suggested to have a role in chromatin remodeling^11^ we were curious whether loss of *His4r* affects the circadian rhythm of flies and performed circadian activity measurements. Circadian rhythm of male *Drosophila* can be characterized by two activity peaks, one in the early hours of the morning and another at dusk, while they are less active in between, with nearly continuous inactivity at night^24^. We compared the daily activity of 1-week-old *His4r*^*rev5*^ and *His4r*^*Δ42*^ male *Drosophila* over a 24 h period of time. Our results show that the loss of *His4r* has no remarkable effect on the sleep pattern (Fig. 3C), total daily movement count (Fig. 3D) or the total amount of sleep (Fig. 3E), suggesting that His4r has no significant role in circadian related chromatin remodeling processes.

### Loss of *His4r* has a mild effect on chromatin structure and transcription

**Figure 4:**
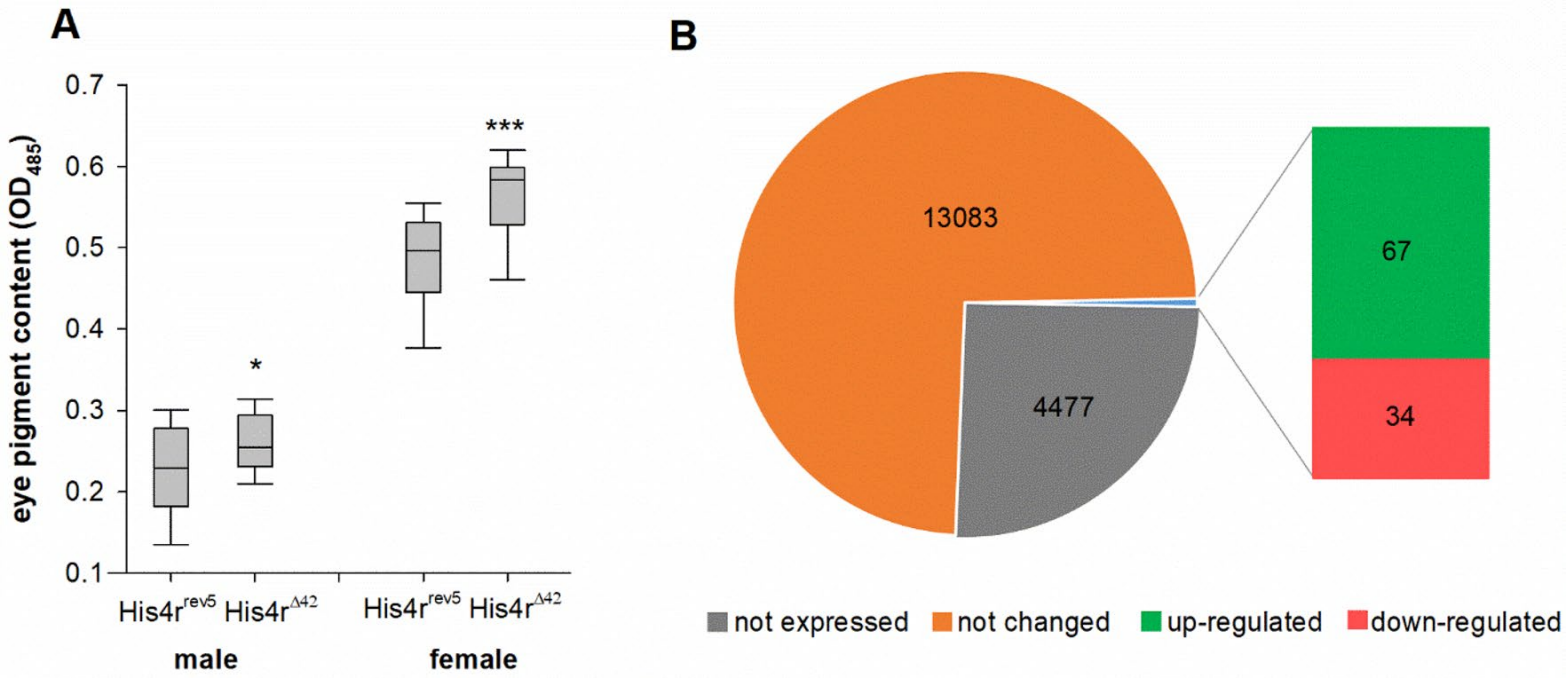
Loss of *His4r* has mild effect on PEV and transcription. **(A)** Heterozygous loss of *His4r* results in mild but significant suppression of position-effect variegation of *w*^*m4h*^ in both genders. Boxplot shows the distribution (first quartile, median, third quartile, and 10th and 90th percentiles as whiskers) of eye pigment absorbance values measured at 485 nm. *P < 0.05, ***P < 10^−3^ (t-test, n = 20). **(B)** Loss of *His4r* has a modest effect on transcriptional activity. Out of the 13,184 genes that were transcriptionally active (FPKM ≥ 1) in males only 101 showed altered expression levels in *His4r*^*Δ42*^ homozygotes compared to *His4r*^*rev5*^ controls: 67 were up- while 34 were down-regulated.

As a histone replacement protein His4r was assumed to have a role in chromatin organization. To test the involvement of His4r in chromatin organization we performed position-effect variegation (PEV) analysis. When an euchromatic gene is juxtaposed with heterochromatin by rearrangement or transposition it causes its transcriptional silencing in some of the cells in a stochastic pattern. As this phenotype is the consequence of a change in the position of the gene in the genome, rather than a change in the gene itself, this phenomenon was named “position-effect variegation”. For PEV analysis we applied the variegating phenotype of *white-mottled* (*w*^*m4*^) allele, which is characterized by white spots in the otherwise red eyes. The whiter the eye the higher the rate of silencing^25^. Our results show that compared to *His4r*^*rev5*^ controls heterozygous loss of *His4r* results in ~ 15% higher eye pigment concentration both in males (OD_485_ values: *His4r*^*rev5*^ 0.2276 ± 0.06 and *His4r*^*Δ42*^*/* + 0.2623 ± 0.05, P = 0.04452, t-test) and in females (OD_485_ values: *His4r*^*rev5*^ 0.483 ± 0.04 and *His4r*^*Δ42*^*/* + 0.55985 ± 0.05, P = 0.00043, t-test) (Fig. 4A). According to our findings *His4r*^*Δ42*^ acts as a mild dominant PEV suppressor, suggesting that His4r might have a role in the formation or maintenance of condensed chromatin structure.

Next, we aimed to assess whether loss of *His4r* that affects chromatin structure also influences transcriptional activity. For this we compared the transcriptome of 7-days-old adult *His4r*^*Δ42*^ and *His4r*^*rev5*^ males by poly(A) RNA-sequencing (RNA-seq). We found that out of the 17,661 transcriptional units listed in *Drosophila melanogaster* gene annotation release r6.13 13,184 was expressed (using cut-off level of FPKM ≥ 1) in at least one of the genotypes, 12,572 was expressed in both genotypes, and 11,207 had sequencing read counts and distribution suitable for statistical testing. We found that the transcriptome profile of *His4r*^*Δ42*^ flies was very similar to that of the *His4r*^*rev5*^ revertant control with high correlation (r = 0.995) and median transcript levels of 7.915 FPKM (Fragments Per Kilobase of transcript per Million mapped reads) in the case of *His4r* mutants and 7.826 FPKM in the case of the revertants. In *His4r* mutants the transcript levels of 101 genes (0.9% of statistically tested, 0.57% of total) was significantly (false discovery rate q < 0.05) altered: 67 was up-regulated while 34 was down-regulated (Fig. 4B). Of note, *His4r* was highly expressed in *His4r*^*rev5*^ (FPKM = 336.95) while it was below the expression cut-off (FPKM = 0.46) in *His4r*^*Δ42*^, functionally validating both the revertant and the deletion mutant lines. Based on Gene Onthology analysis most up-regulated genes are associated with GO Biological Process terms of cellular process (GO:0009987), metabolic process (GO:0008152) or localization (GO:0051179) (Supplementary Fig. S3), while most down-regulated genes are associated with terms cellular process (GO:0009987), multicellular organismal process (GO:0032501) or metabolic process (GO:0008152) (Supplementary Fig. S4). The most dysregulated genes both among the up-regulated and down-regulated ones are associated with GO Molecular Function categories catalytic activity (GO:0003824) and binding (GO:0005488) (Supplementary Figs. S5 and S6). However, no molecular function or biological process terms were overrepresented in these groups at a statistically significant level.

### *His4r*^*Δ42*^ flies show increased heat-stress resistance with stronger induction of heat shock protein genes and less condensed chromatin at the *Hsp27* promoter

**Figure 5:**
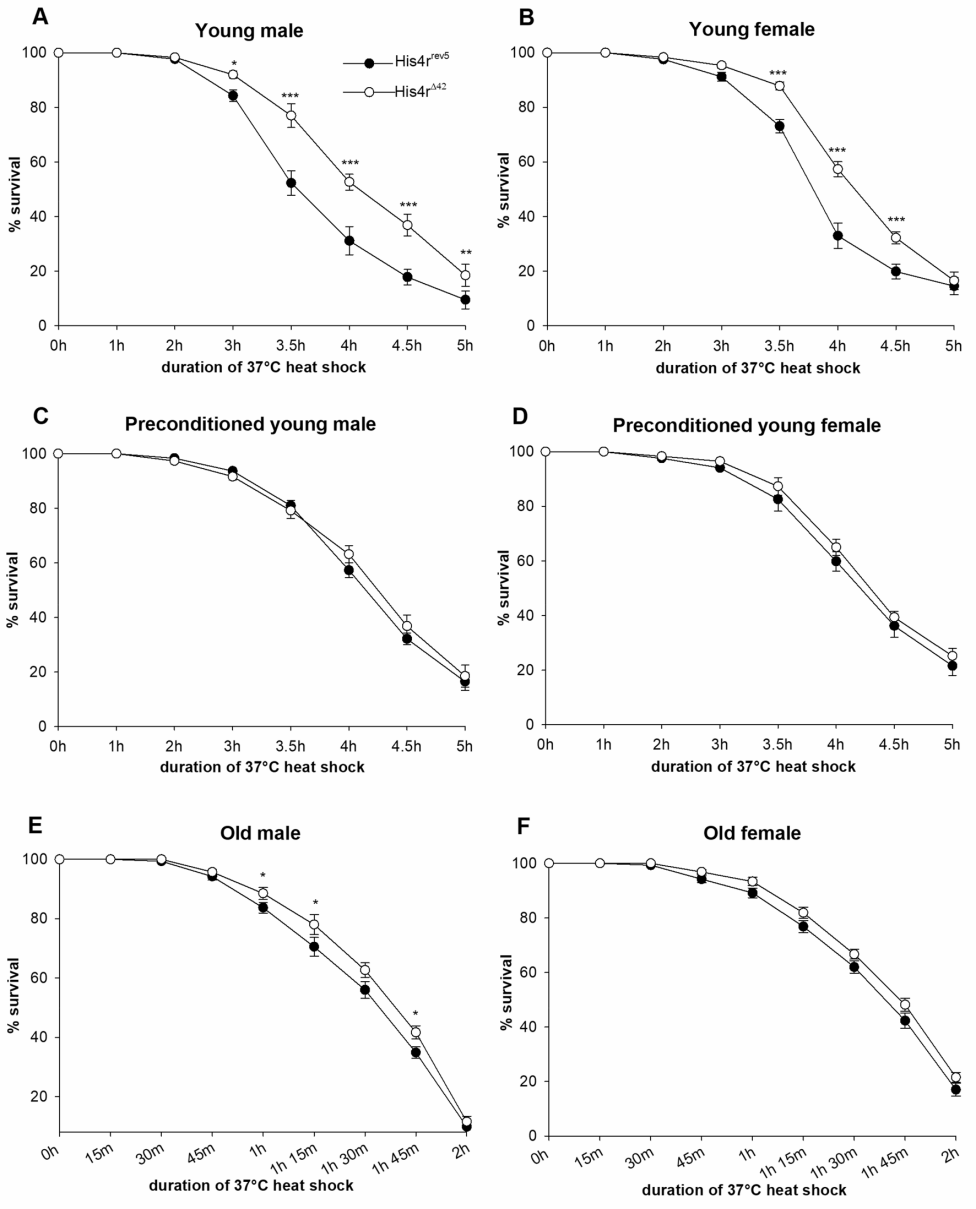
Loss of *His4r* increases heat-stress tolerance. Both 3–5 days old *His4r*^*Δ42*^ males (**A**) and females (**B**) showed increased heat-stress tolerance compared to revertant controls. The survival rate of *His4r*^*Δ42*^ males was significantly higher than controls after 3.5–5 h of heat-stress at 37 °C, while the survival rate of *His4r*^*Δ42*^ females was higher than controls after 3.5–4.5 h of heat-stress. After preconditioning for 30 min at 34 °C on two preceding days there was no difference between the survival rates of 3–5 days old *His4r*^*Δ42*^ males (**C**) or females (**D**) and corresponding controls after heat-stress at 37 °C. 30 days old males (**E**) and females (**F**) showed reduced heat-stress tolerance compared to younger flies (**A**,**B**), and homozygous loss of *His4r* had only a minor effect on survival following heat shock (male: P = 0.009; female: P = 0.0114, log-rank test). The graph shows the average survival rate, error bars show standard error, n = 30 vials (20 flies/vial).*P < 0.05, **P < 10^−2^, ***P < 10^−3^, Mann–Whitney U test.

Next, we aimed to test whether His4r plays a role in the response to environmental stress. For this, we chose to analyze response to heat-stress, a well characterized paradigm in chromatin dynamics studies. First, we exposed 3–5 days old *His4r*^*Δ42*^ and *His4r*^*rev5*^ flies to 37 °C heat shock for 0–5 h periods and determined their survival rates after specific lengths of heat-stress (0, 1, 2, 3, 3.5, 4, 4.5, and 5 h). We found that flies started to perish after 3 h of heat shock treatment and by 5 h only ~ 20% was alive (Fig. 5A,B). The survival rates of both *His4r*^*Δ42*^ males (P < 1.0 × 10^–10^, log-rank test) and females (P = 4.4 × 10^–8^, log-rank test) was higher compared to the *His4r*^*rev5*^ controls after 3.5 h of heat shock (Fig. 5A,B). Next, we tested the effect of preconditioning for 30 min at 34 °C for 2 consecutive days prior to heat shock treatment at 37 °C on the survival of 3–5 days old flies. We found that in these experiments flies started to perish after 3.5 h of 37 °C heat shock and by 5 h of treatment only ~ 20% percent was alive. Interestingly, after preconditioning there was no significant difference between the survival rates of *His4r*^*Δ42*^ and *His4r*^*rev5*^ control flies, both groups had survival rates similar to that of *His4r*^*Δ42*^ without preconditioning (Fig. 5C,D). We also performed the heat shock response experiment with 1-month-old flies that were exposed to 37 °C heat shock for 0–120 min in 15 min intervals. These older flies were more sensitive to heat-stress, they started to perish after 1 h of 37 °C heat shock treatment and by 2 h of treatment only ~ 10–20% percent was alive both in the case of *His4r*^*Δ42*^ and *His4r*^*rev5*^ control males and females. The survival of both *His4r*^*Δ42*^ males (P = 0.009, log-rank test) and females (P = 0.0114, log-rank test) was only slightly better than the survival of corresponding control flies (Fig. 5E,F).

**Figure 6:**
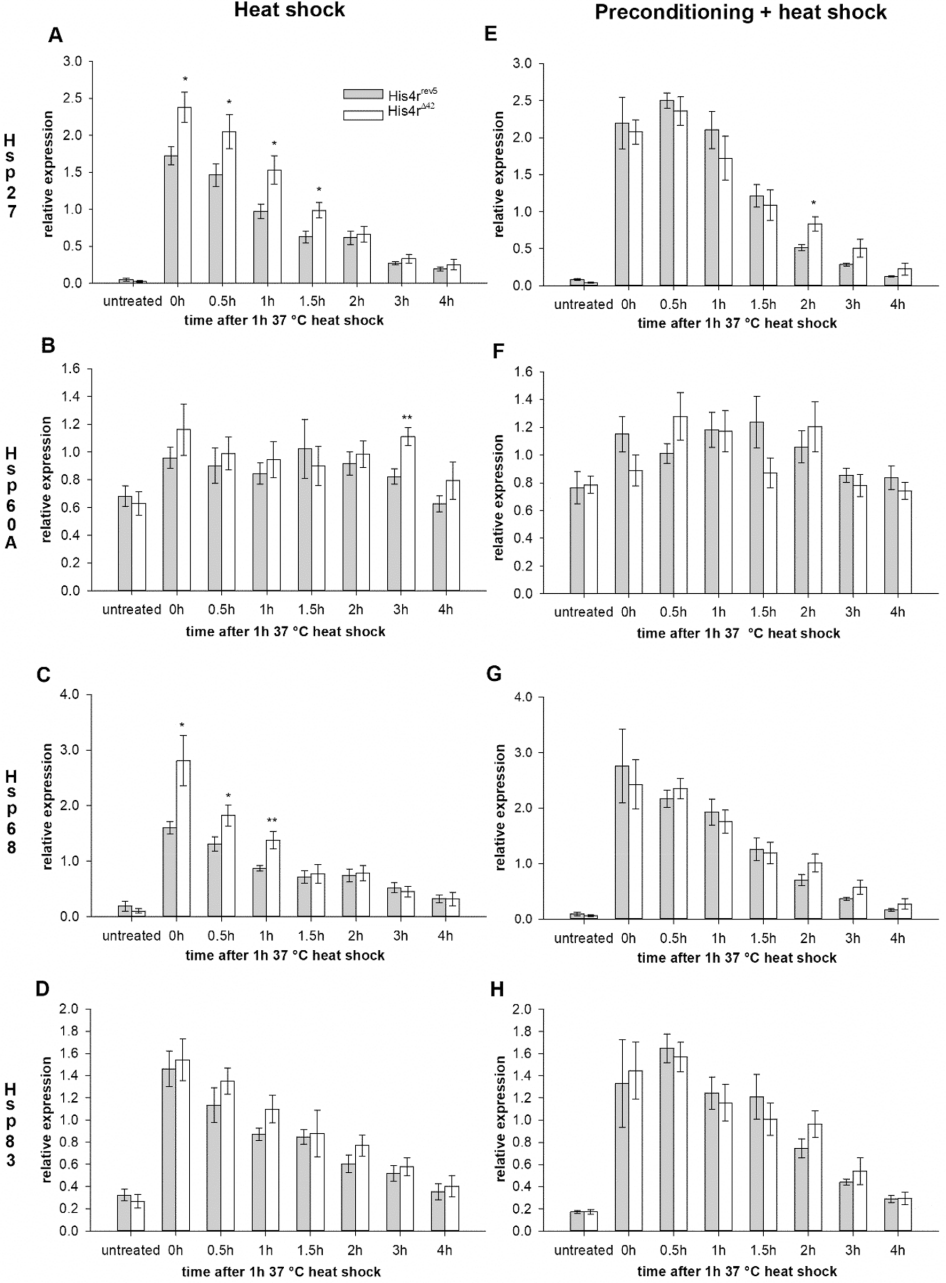
His4r affects HSP gene induction after heat shock. The transcript levels of *Hsp27* (**A**), *Hsp68* (**C**) and *Hsp83 * (**D**) HSP genes showed dramatic increase after 37 °C heat shock (P < 0.001, Kruskal–Wallis Test) both in *His4r* mutants (white) and controls (grey) that gradually decreased during the post-heat shock incubation period, while there was no change in the expression of *Hsp60A* (**B**). However, following heat shock the transcript levels of *Hsp27* **(A)** and *Hsp68* **(C)** were significantly higher in *His4r* mutants than in controls. (**E–H**) After preconditioning at 34 °C the expression of HSP genes in *His4r*^*rev5*^ controls reaches the same elevated levels as in *His4r*^*Δ42*^ flies. Asterisks mark significant differences between *His4r*^*Δ42*^ and *His4r*^*rev5*^ samples, *P < 0.05, **P < 10^−2^, Mann–Whitney U test.

We hypothesized that increased heat-stress resistance in *His4r* mutants might be the consequence of increased expression of Heat shock protein (HSP) genes due to altered chromatin structure at regulatory elements. For testing we selected representative genes from each HSP family (*Hsp27*, *Hsp60A*, *Hsp68* and *Hsp83*) that showed the highest expression upon heat shock according to modENCODE data^14^. We measured gene expression before heat shock and 0, 0.5, 1, 1.5, 2, 3, or 4 h after a 1 h long 37 °C heat shock treatment by RT-qPCR and detected dramatic increase in the transcript levels of *Hsp27*, *Hsp68* and *Hsp83* after heat shock (P < 0.001 in each case both in *His4r*^*Δ42*^ and *His4r*^*rev5*^ flies, Kruskal–Wallis Test) that gradually decreased during the post-heat shock incubation period (Fig. 6A,C,D), while *Hsp60A* showed a much milder (statistically not significant) response (Fig. 6B). We found that the induction of *Hsp27* (Fig. 6A) and *Hsp68* (Fig. 6C) was significantly stronger in *His4r*^*Δ42*^ than in control flies, while the induction of *Hsp83* (Fig. 6D) was not significantly different between the two genotypes. It is worth to note that despite stronger gene induction in *His4r*^*Δ42*^ flies the passing off of transcript levels showed similar patterns in the two genotypes and evened up after 1.5 h in the case of *Hsp68* and after 2 h in the case of *Hsp27*. We were interested to know whether the differences in the induction of HSP genes are cancelled out after preconditioning similarly to what we had observed in the case of post-heat shock survival rates. Therefore, we repeated the previous experiment after preconditioning flies for 2 days at 34 °C for 30 min daily. After 37 °C heat shock treatment the induction of HSP genes in *His4r*^*Δ42*^ flies was very similar to the levels we measured without preconditioning. However, in *His4r*^*rev5*^ controls the induction of HSP genes was more pronounced when the flies were preconditioned. Consequently, when preconditioning was applied HSP gene expression levels of *His4r*^*Δ42*^ and *His4r*^*rev5*^ control flies became alike and reached similar elevated levels to what we had observed in *His4r*^*Δ42*^ without preconditioning (Fig. 6E–H). Thus, preconditioning cancels out the differences both in survival rates and HSP transcript levels between *His4r* mutant and control flies.

**Figure 7:**
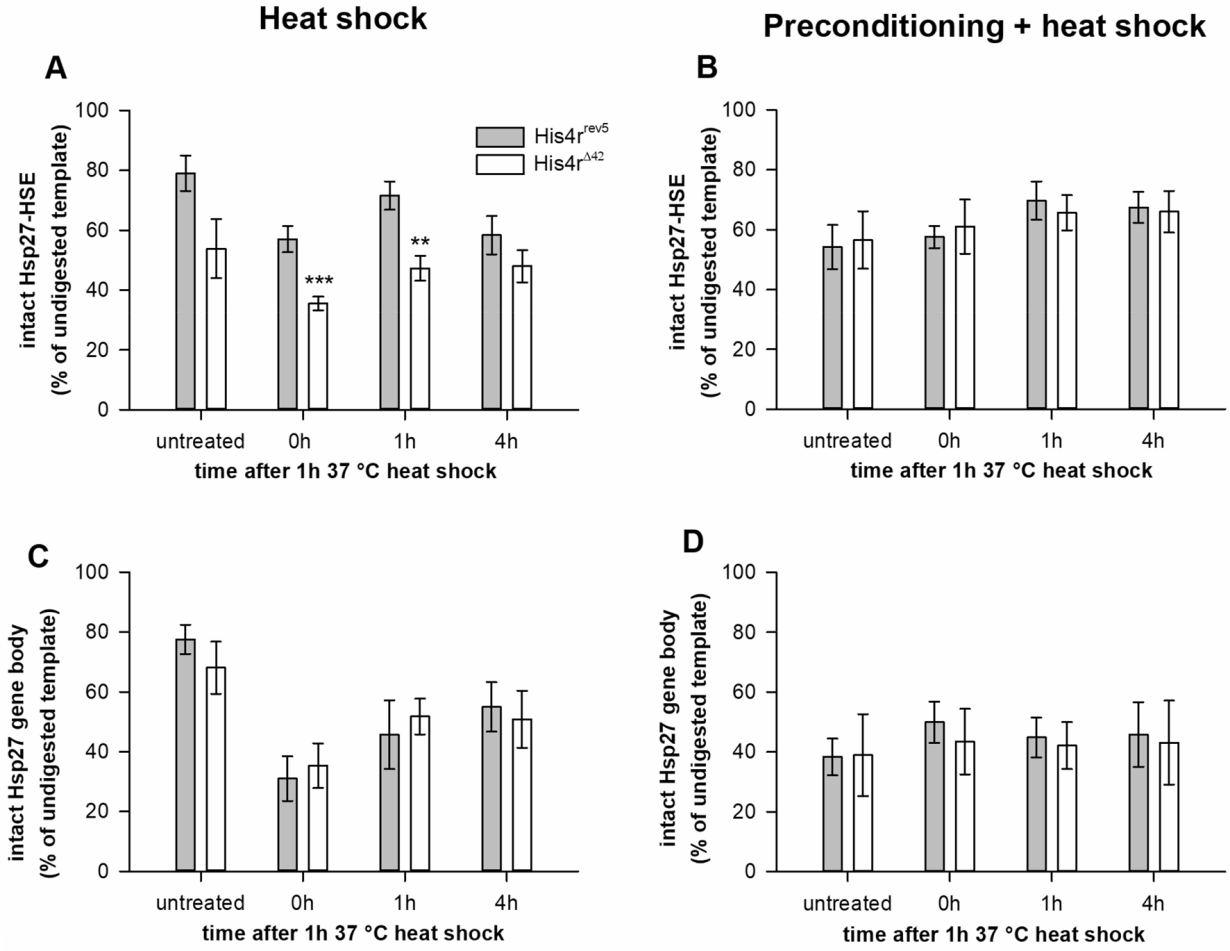
His4r is a component of chromatin at the HSE containing regulatory region of *Hsp27*. **(A)** In *His4r* mutants (white) lower amount of intact Hsp27-HSE was present after MNase digestion even without heat shock treatment compared to the controls, although this difference was not significant. Following heat shock significantly less PCR products were detected in *His4r*^*Δ42*^ flies than in controls indicating more open chromatin.in the absence of His4r. **(B)** After preconditioning for 2 days in the controls the amount of intact Hsp27-HSE was reduced to the levels of *His4r* mutants cancelling out the difference between the two genotypes, furthermore, heat shock did not result in further chromatin opening. Chromatin accessibility at the gene body of *Hsp27* in heat shock experiments without preconditioning **(C)** or with preconditioning **(D)** did not differ between *His4r* mutants and controls. **P < 10^−2^, ***P < 10^−3^, t-test.

Next, to test our hypothesis that His4r plays a role in the regulation of HSP genes by influencing local chromatin structure we performed chromatin accessibility assays. We analyzed the accessibility of the regulatory region containing three heat shock elements (HSE) upstream of the *Hsp27* promoter^26^ and the accessibility of the coding sequence of the gene by performing qPCR on MNase digested chromatin templates prepared from flies treated as in the previous experiments. In these experiments loss of nucleosomes allows the access of MNase to DNA that leads to less PCR products while the presence of intact nucleosomes protect DNA from MNase digestion and leads to more PCR products. 37 °C heat shock induced loss of nucleosomes both at the HSE containing regulatory region (*His4r*^*rev5*^ P = 0.01199; *His4r*^*Δ42*^ P = 0.0375, t-test) (Fig. 7A) and at the gene body (*His4r*^*rev5*^ P = 0.00098; *His4r*^*Δ42*^ P = 0.02244, t-test) (Fig. 7C) that was detected by lower amount of intact DNA sequences after MNase treatment. In *His4r*^*Δ42*^ samples we measured lower amount of intact Hsp27-HSE elements after MNase digestion than in *His4r*^*rev5*^ controls even before heat shock treatment, however this difference was not significant (P = 0.07714, t-test) (Fig. 7A). Immediately after the 1-h 37 °C heat shock treatment significantly less PCR product was detected in *His4r*^*Δ42*^ flies compared to the *His4r*^*rev5*^ controls (P = 0.00053, t-test) (Fig. 7A) indicating that the chromatin structure of the Hsp27-HSE site was more accessible in the mutant. The amount of intact Hsp27-HSE templates in *His4r*^*Δ42*^ remains significantly lower (P = 0.0022, t-test) than in controls 1 h after the heat shock (Fig. 7A), however, this difference levels off 4 h after heat shock treatment (Fig. 7A). When we preconditioned *His4r*^*Δ42*^ and *His4r*^*rev5*^ flies for 2 days at 34 °C we observed similar quantities of Hsp27-HSE specific PCR amplicons before heat shock in both genotypes (Fig. 7B) as in *His4r*^*Δ42*^ flies without preconditioning (Fig. 7A). This result indicates that preconditioning relaxed chromatin at the HSEs in the control while did not have an effect in the *His4r* mutant. Furthermore, preconditioning before heat shock treatment abolished the difference in the amount of intact HSP27-HSE elements between *His4r*^*Δ42*^ and *His4r*^*rev5*^ controls at all inspected time points (Fig. 7B) similarly to what we had observed previously in the case of survival rates and HSP gene expression.

To investigate whether the effect of lack of His4r is specific for the regulatory region of *Hsp27* or also influences chromatin accessibility at the gene body we performed qPCRs on the same samples using *Hsp27* coding sequence specific primers. Our results show that in heat shock experiments there is no difference in the accessibility of chromatin at the *Hsp27* gene body between *His4r*^*Δ42*^ mutants and *His4r*^*rev5*^ controls neither without (Fig. 7C) or with preconditioning (Fig. 7D). This suggests that while His4r is a significant component of chromatin at the regulatory region of *Hsp27* it does not play an important role at the gene body.

## Discussion

The packaging of DNA into chromatin structure presents a barrier to nuclear processes like replication, gene expression or DNA repair. One way of loosening condensed chromatin structure or altering chromatin state is through the replacement of canonical nucleosomal core histones with histone variant proteins^5^. Most histone variants can be associated with specific chromatin states or chromatin related processes, for example H2A.X is involved in DNA double-strand break repair, while H3.3 is deposited to transcribed genes^6^. In higher eukaryotes variants of all core histones but H4 exist. Histone H4 is a slowly evolving protein which shows divergence in some protozoan taxa but sequence variants in higher eukaryotes have not been identified yet^27^. In the *Drosophila* genus a single-copy gene variant of H4, *His4r*, exists that is highly conserved in all 14 *Drosophila* species analyzed^11^. The amino acid sequence of His4r proteins is identical to that of H4 in most *Drosophila* species including *D. melanogaster* making His4r rather a replacement histone than a variant^10,11^. However, *His4r* genes have different regulation and gene structure than canonical, replication-dependent *H4* genes located in the histone gene cluster^10,11^. These features suggest that His4r might play a conserved role in chromatin remodeling but its specific functions must depend on the regulation of its expression.

In the *His4r*^*Δ42*^ mutant we generated the entire coding region is deleted making it an amorphic (genetic null) allele. Similarly to carriers of loss of function mutations of histone H3 variants *His3.3A* or *His3.3B*^28,29^ homozygous *His4r*^*Δ42*^ mutants proved to be viable without any obvious morphological defects. Furthermore, in contrast to the sterility caused by complete loss of H3.3 in *H3.3B; H3.3A* double mutants^28,29^ both genders are fertile in the absence of His4r. We observed mild (~ 32%) sublethality of *His4r* mutant animals that occurred in part before pupariation and in part during metamorphosis. Remarkably, the viability of *His4r*^*Δ42*^ females was lower than that of male siblings and this disproportional loss of females occurred before pupariation during the embryonic and/or larval developmental stages. There are at least two sex specific processes that might be responsible for this phenomenon: sex determination and dosage compensation. The key factor in *Drosophila* sex determination is the female specific RNA binding protein, sex lethal (SXL)^30^. The maintenance of sex specific *Sxl* expression is based on autoregulatory alternative splicing that leads to the production of functional SXL proteins in females and a truncated, inactive SXL in males. However, the initiation of *Sxl* expression is primarily a transcriptional response, which is regulated by the expression levels of specific X-encoded proteins that serve to relay the dose of the X chromosome^30^ and also influenced by local chromatin environment of the female specific early *Sxl*_*Pe*_ promoter^31^. In males in the absence of SXL the Male Specific Lethal (MSL) complex is formed and dosage compensation is activated^32^. MSL increases the transcriptional output from the single X chromosome by acetylation of lysine 16 on histone H4. Loss of dosage compensation in males and its activation in females are both lead to lethality^32^. Thus, both sex determination and dosage compensation are influenced by chromatin structure and thus, might be influenced by the availability of histones, such as His4r. However, whether loss of His4r leads to more pronounced female sublethality by disturbing these processes is yet to be determined. We also investigated the effects of loss of His4r on adult phenotypes like daily activity or aging but we observed no remarkable defects. These data together suggest that the functions of His4r are mostly but not fully redundant and might be substituted by H4 or the processes it is involved in are not essential under standard environmental conditions.

As a histone protein His4r is expected to have a role in chromatin organization. During the S phase of the cell cycle the large amounts of histones required for chromatin replication are supplied by replication-dependent genes of the histone gene cluster. However, histones are also deposited to chromatin during the interphase. The histone H3.3 variant is known to be incorporated into chromatin in a replication independent manner and to replace H3 on transcriptionally active genes^33,34^ and also at silenced heterochromatic regions^35^ by the histone regulator A (HIRA)^36^ and death-associated protein (DAXX)/α-thalassemia X-linked mental retardation protein (ATRX)^35^ histone chaperon complexes, respectively. In mice, this process leads to the accumulation of H3.3 at near saturation levels (~ 99% of total H3) in liver, kidney and brain cells by late adulthood^37^. As the HIRA and DAXX/ATRX histone chaperone complexes bind H3.3-H4 dimers^38^ histone replacement requires equimolar amounts of H3.3 and H4. We assume that His4r as a replication independent histone is a partner of H3.3 in this process and might have similar effects on chromatin organization and transcription. To investigate these assumptions, we performed position-effect variegation (PEV) and transcriptomic analysis. Our data show that partial loss of *His4r* acts as a mild PEV suppressor, suggesting that it contributes to the formation or maintenance of condensed chromatin structure. Importantly, loss of function mutations of DLP, the *Drosophila* homolog of the heterochromatic H3.3-H4 specific histone chaperone DAXX, also suppress PEV^35^. In transcriptomic analysis we found that only a small subset of genes showed significantly altered transcriptional activity in *His4r*^*Δ42*^ male flies and this set was not enriched for genes specific for any biological process. The general trend of gene expression changes in the absence of His4r (67 genes up-regulated and 34 genes down-regulated) was similar to what was observed by others in the absence of H3.3 (288 up- and 99 down-regulated genes)^28^ i.e. in accordance with their role in chromatin formation up-regulation of transcriptional activity was more common in the absence of either His4r or H3.3.

As the loss of *His4r* has no remarkable adult phenotypes and only mild effects on transcription under standard conditions we decided to test its potential role in environmental stress response. For this we chose to analyze the response to heat shock that is commonly used in chromatin dynamics studies. Upon heat stress Heat Shock Factor (HSF), the *Drosophila* homologue of the mammalian heat stress responsive transcriptional factor HSF1, binds to its recognition sequence (Heat Shock sequence Elements, HSE) in trimeric form and elicits complex transcription regulatory steps^39^. The presence of HSEs is not sufficient to HSF binding in vivo, however, it is also highly dependent on the local chromatin environment. Genome-wide ChIP-seq analysis showed that HSF was present at less than 15% of HSE elements in the genome after heat shock and it co-localized with marks of active chromatin including acetylated H3 and H4, trimethylated H3K4, dimethylated H3K79 and mono-ubiquitylated H2B^40^. Heat shock protein (HSP) genes whose products function as molecular chaperones are well characterized targets of HSF. The molecular events of HSP activation in flies are described in most detail in the case of *Hsp70* and *Hsp90* genes. Upon heat shock HSF releases RNA polymerase II from promoter-proximal pausing with the help of transcription elongation factors and in interaction with nucleosome remodeling factors and histone modifying enzymes reorganizes chromatin by inducing H2K5 and H4 specific acetylation and ADP ribosylation of histones and eviction of nucleosomes^39,41^. Consequently, active transcription results in the disassembly of nucleosomes on HSP genes. After heat shock, on nucleosome depleted *Hsp70* genes H3.3 histone variant containing nucleosomes are assembled with the aid of XNP chromatin remodeler and HIRA and ASF1 histone chaperones^36^.

We performed heat shock experiments with *His4r* mutant adults and found that the survival rate of young *His4r*^*Δ42*^ flies is higher compared to the control upon 37 °C heat shock treatment. 34 °C preconditioning increased the survival rate of control flies to the level observed in the *His4r* mutant strain without preconditioning but it did not cause notable change in the survival rate of *His4r* mutants. Furthermore, in contrast to the significantly improved stress resistance that we observed in the case of young adults the survival rate of 1-month-old *His4r*^*Δ42*^ flies was only slightly better compared to the control, which probably results from the loss of *His4r* with age as based on our data its transcriptional activity gradually declines during aging. These results suggest that the higher survival rate of *His4r*^*Δ42*^ flies after heat shock might be the consequence of a less condensed chromatin structure in the absence of His4r that can be also achieved by preconditioning or aging. Preconditioning and older age abolishes the positive effects of loss of *His4r* on heat stress tolerance because on the one hand preconditioning might lead to the restructuring of chromatin while on the other hand in older adults the supply of available His4r is limited.

We measured the transcriptional activity of different HSP genes to ascertain whether altered HSP expression coincides with the above described effects in the lack of His4r. Heat shock treatment at 37 °C provoked increased induction of *Hsp27* and *Hsp68* in the absence of His4r that might be responsible for the higher survival rate of *His4r*^*Δ42*^ flies. However, preconditioning at 34 °C abolished the difference between *His4r* mutant and control flies by raising HSP gene expression levels of the controls to the levels observed in *His4r *^*Δ42*^ flies, while causing no notable transcriptional change in the *His4r*^*Δ42*^ strain itself. In conclusion, the increased survival rate of young *His4r*^*Δ42*^ flies after heat shock goes hand in hand with stronger induction of some HSP genes. However, preconditioning and old age cancels these effects suggesting that preconditioning, age and the absence of His4r might exert similar effects on local chromatin structure.

To further confirm our hypothesis we performed chromatin accessibility assays both at the regulatory region and at the gene body of the *Hsp27* gene in heat shock experiments with or without preconditioning. In the *Hsp27* promoter three HSEs are located between − 370 and − 270 upstream of the transcriptional start site^26^. The sequence between the TATA box and the HSEs are organized by a positioned nucleosome^42^ and is dispensable for heat induction in vitro^26^*.*Our results show that heat shock induces nucleosome remobilization both at the HSE containing regulatory region and at the gene body of *Hsp27*, however, loss of *His4r* only affects chromatin accessibility at the HSEs. Although upon heat shock chromatin is more accessible at the *Hsp27* promoter in the absence of His4r, preconditioning cancels this effect which is in concordance with increased post-heat shock survival and HSP gene expression induction. Interestingly, our results indicate that chromatin accessibility at the gene body is not affected by His4r as no difference was observed between *His4r* mutant and control flies.

In conclusion, our findings suggest that although His4r is a non-essential protein in fruit flies it might play a role in fine tuning the transcriptional response to environmental stress conditions by participating in the dynamic formation of local chromatin structure at regulatory regions of inducible genes.

## Materials and methods

### Fly stocks and crosses

Fly stocks were maintained and crosses were done on standard *Drosophila* medium at 25 °C unless otherwise noted. The *y*^*1*^* w*^*67c23*^*; P{EPgy2}His4r*^*EY06726*^ line was from the Bloomington Drosophila Stock Center, y^*^ w^*^; P{w[+ *mW.hs]* = *FRT(w[hs])}2A P{ry[*+ *t7.2]* = *neoFRT}82B PBac{SAstopDsRed}LL05512 P{y[*+ *t7.7] ry[*+ *t7.2]* = *Car20y}96E / TM6B, Tb*^*1*^ was from the Kyoto Stock Center (DGRC).

To generate *His4r* deletion lines *y*^*1*^* w*^*67c23*^*; P{EPgy2}His4r*^*EY06726*^ males were mated with *w; Dr Δ2-3/TM3 Δ2-3* females, then *w; P{EPgy2}His4r*^*EY06726*^*/TM3 Δ2-3* male jumpstarter F1 offspring were crossed with *w; TM2/TM6* females. *w; His4r*^*revertant*^*/TM2* revertant male F2 progeny were selected based on the white eye color appearing as a result of the loss of the P{EPgy2} element and crossed individually to *w; TM2/TM6* females. Five days after mating founder *w; His4r*^*revertant*^*/TM2* F2 males were removed from the vials and were genotyped by PCR while their progenies were used to establish *w; His4*^*revertant*^ lines.

### Deletion analysis by PCR

Individual *w; His4r*^*revertant*^*/TM2* male candidates were homogenized in 50 μl squashing buffer (10 mM Tris–HCl, 1 mM EDTA, 25 mM NaCl, 200 ng/μl Proteinase K) and incubated for 60 min at 37 °C followed by 15 min at 85 °C. 4 μl homogenates were used as templates in 20 μl PCR reactions using DreamTaq DNA polymerase (Thermo Fisher Scientific, TFS) with His4rFseq forward and His4rgR or His4rE3CR (Supplementary Table S1) reverse primers at 250 nM concentration. *His4r* deletions were identified based on the presence of PCR products shorter than with *wild-type* control template. Sanger capillary sequencing was performed on purified His4rFseq—His4rgR PCR amplicons with His4rFseq sequencing primer by Delta Bio 2000 Ltd. (Szeged, Hungary).

### Complementation test and fecundity assay

*w; His4r*^*Δ42*^ (henceforth *His4r*^*Δ42*^) virgins were mated to y^*^ w^*^; P{w[+ *mW.hs]* = *FRT(w[hs])}2A P{ry[*+ *t7.2]* = *neoFRT}82B PBac{SAstopDsRed}LL05512 P{y[*+ *t7.7] ry[*+ *t7.2]* = *Car20y}96E / TM6B, Tb*^*1*^ (henceforth *His4r*^*LL05512*^)^20^ males to generate *w; His4r*^*LL05512*^*/His4r *^*Δ42*^ progeny. *w; His4r*^*LL05512*^*/His4r *^*Δ42*^ males and females were crossed separately with *w*^*1118*^ flies then the vials were checked for the presence of viable progeny.

To measure the fecundity of homozygous *His4r*^*Δ42*^* and His4*^*rev5*^ flies freshy eclosed (0–24 h old) males and females were collected and mated for 3 days on yeast rich medium before the experiment. 10 parallel crosses were made per genotype, with five females and five males in each vial. Flies were passed to a fresh vial every day for 5 days and the number of eggs laid were counted in each vial. Fecundity is expressed as the average number of eggs laid per female per day ± standard error of mean (SEM).

### Viability assays

To determine the eclosion rate of *His4r*^*Δ42*^ flies a two-step crossing scheme was performed. Homozygous *His4r*^*Δ42*^ virgins were mated with *M{3xP3-RFP.attP}ZH-86Fb* males (a homozygous viable strain commonly used as a docking site for φC31 integrase mediated transgenesis, henceforth called 86Fb), then heterozygous *His4r*^*Δ42*^/86Fb males were backcrossed to *His4r*^*Δ42*^ virgins in 30 parallel vials. The number of homozygous and heterozygous progeny was determined based on the presence of the RFP marker. Data is shown as the total number of eclosed flies, for statistical analysis binomial test and Student’s t-test were used.

To determine the ratio of male and female progeny during the third larval stage freshly eclosed *His4r*^*Δ42*^ or *His4r*^*rev5*^ males and females were mated for 3 days on yeast rich medium, then sorted to 20 vials (5 males and 5 females each) per genotype. Wandering 3rd instar larvae (wL3) were collected and sorted under a stereo microscope where male and female larvae can be distinguished by their gonads. Sorted larvae were placed on fresh vials where pupation and eclosion analysis was carried out. Data is shown as mean ± standard error of mean (SEM), for statistical analysis Student’s t-test was used.

### Longevity assay

Freshly eclosed *His4r*^*rev5*^ and *His4r*^*Δ42*^ males and females were collected separately (30 flies/ vial). To determine longevity, flies were kept at 25 °C, transferred to fresh vials every second day and the number of deceased individuals was recorded daily. At least 240 flies per genotype were used for longevity analysis. For statistical analysis OASIS 2 (Online Application for Survival Analysis 2) software was used^43^.

### Daily activity measurement

Freshly eclosed *His4r*^*rev5*^ and *His4r*^*Δ42*^ males were collected and kept at 25 °C (30 flies/vial). Flies were synchronized and entrained by exposing them to 12:12 h light (~ 250 lx):dark (LD) cycles for 1 week before performing the activity recordings with DAM2 Drosophila Activity Monitor (TriKinetics Inc, Waltham, MA, USA) that records the activity of 32 individual flies simultaneously. We recorded the daily activity of flies over a period of 24 h starting from ZT0 (Zeitgeber Time: refers to time in hours during a light–dark cycle where ZT0 = lights on and ZT12 = lights off). Data were collected with DAMSystem3 for Windows and analyzed using pySolo analysis software^24^ and by Excel functions. Data are presented as mean ± standard error of mean (SEM).

### PEV analysis

*His4r*^*rev5*^ and *His4r*^*Δ42*^ males were mated with In(1)w^m4h^ females (in which a chromosomal inversion places the *white* gene next to the pericentric heterochromatin^44^) to produce progeny. Variegation of the *white* gene was quantitated by measuring eye pigment content. To extract eye pigments 20 fly heads were homogenized in 1.2 ml volume of 1:1 mixture of chloroform and 0.1% ammonium-hydroxide. After phase separation by centrifugation at 13 kRPM for 3 min the upper water phase containing the pigments was collected and its optical density was measured at 485 nm in a spectrophotometer. Data are presented as mean ± standard error of mean (SEM), for statistical analysis Student’s t-test was used.

### RNA-sequencing and data analysis

Total RNA was prepared from 7-day-old adult males (3 biological replicates per genotype, 30 males per replicate) using Trizol Reagent (Invitrogen) and MN NucleoSpin RNA Clean-up kit (Macherey–Nagel). RNA integrity and concentration were determined with Agilent 2100 Bioanalyzer capillary gel electrophoresis using Agilent RNA 6000 Nano kit. Poly(A) RNA fraction from 800 ng total RNA was selected with NEBNext Poly(A) mRNA Magnetic Isolation Module (New England Biolabs, NEB) then strand-specific, indexed RNA-sequencing libraries were prepared using NEBNext Ultra Directional RNA Library Prep Kit for Illumina (NEB). Fragment length distribution and concentration of sequencing libraries were determined with Agilent 2100 Bioanalyzer using Agilent DNA 1000 kit, then after pooling and denaturing libraries were sequenced in an Illumina MiSeq DNA sequencer using MiSeq Reagent Kit V3-150. Paired-end sequence reads were inspected with FastQC then quality trimmed using Trimmomatic v0.33 with options HEADCROP:15 CROP:55 ILLUMINACLIP:TruSeq3-PE.fa:2:30:10:8:true TRAILING:10 SLIDINGWINDOW:4:20 MINLEN:36. Trimmed read pairs were aligned to the *Drosophila melanogaster* reference genome Dmr6.13 using TopHat v2.0.9 spliced read mapper. After sorting and deduplicating binary alignment files with Samtools 0.1.19, differential gene expression analysis was done with Cuffdiff v2.1.1 using *Drosophila* gene annotation release r6.13.gtf. For gene ontology term analysis we applied PANTHER Overrepresentation Test (released 20190711) with GO Ontology database (released 2019-10-08) using Fisher's exact test with FDR correction.

### Heat shock response analysis

Heat shock-response analysis was carried out on both young (3–5 days-old) and on old (1-month-old) *His4r*^*rev5*^ and *His4r*^*Δ42*^ males and females (20 flies per vial, 30 parallel vials per genotype) reared at 25 °C. Young flies were exposed to 37 °C heat shock for 0 min (no-hs controls), 1 h, 2 h, 3 h, 3.5 h, 4 h, 4.5 h, or 5 h while old flies were exposed to 37 °C heat shock for 0 min (no-hs controls), 15 min, 30 min, 45 min, 1 h, 1 h 15 min, 1 h 30 min, 1 h 45 min, or 2 h. After heat shock flies were transferred to fresh vials and the number of survivors was counted the next morning. Preconditioning of flies was carried out by daily heat shocks at 34 °C for 30 min for two consecutive days prior to the 37 °C heat shock treatment. Data are presented as mean ± standard error of mean (SEM), for statistical analysis OASIS 2 (Online Application for Survival Analysis 2) software and Mann–Whitney U test were used.

### Gene expression analysis by RT-QPCR

For validation of proper gene expression in *His4r*^*rev5*^ and loss of expression in *His4r*^*Δ42*^ flies 7-days-old *Oregon-R*, *His4r*^*rev5*^ and *His4r*^*Δ42*^ males were collected.

For analysis of age dependent *His4r* expression freshly eclosed *w*^*1118*^ wild-type male flies were collected (30 flies/ vial) and kept at 25 °C, sampling times were the following: 1 day, 3 days, 1, 2, 4, 6 and 8 weeks.

For analysis of HSP gene induction 3–5 days-old *His4r*^*rev5*^ and *His4r*^*Δ42*^ males were exposed to 1 h 37 °C heat shock treatment. Samples were collected before treatment and at 0 min, 30 min, 1 h, 1.5 h, 2 h, 3 h and 4 h post-treatment. Preconditioning of flies was carried out at 34 °C for 30 min for two consecutive days prior to heat shock treatment.

Total RNA was isolated from heads (at least 3 biological replicates per sampling time, 20 males per replicate) using Trizol Reagent (Invitrogen). RNA concentration and purity were determined by spectrophotometric measurement with NanoDrop ND-1000 instrument. First-strand cDNA was prepared from 400 ng total RNA after DNaseI (TFS) treatment using TaqMan Reverse Transcription Reagents (TFS) with random hexamer primers following the recommendations of the manufacturer. The resulting cDNA was diluted 1:5 and used for qPCR by the SYBR green method with Luminaris Color HiGreen qPCR Master Mix in a PikoReal Real-Time PCR System (TFS). qPCR based expression data gained with *His4r*, *Hsp27, Hsp60A, Hsp68 and Hsp83* gene specific primers (Supplementary Table S1) were normalized to the expression level of *α-Tubulin at 84B* (CG1913)^45^ housekeeping gene (Supplementary Table S1). Data are presented as mean ± standard error of mean (SEM). For statistical analysis of *His4r* measurements One-Way ANOVA (OWA) with Tukey HSD post-hoc test was performed, while Kruskal–Wallis test and Mann–Whitney-U test was used in case of HSP genes.

### Chromatin accessibility assay

#### Isolation of nuclei

Chromatin accessibility assays were carried out on 3–5 days-old *His4r*^*rev5*^ and *His4r*^*Δ42*^ flies. Flies were exposed to 1 h 37 °C heat shock treatment. Samples were collected before treatment and at 0 h, 1 h, and 4 h post-treatment. Nuclei were isolated from at least 3 biological replicates per sampling time, ~ 500 flies per replicate. Flies were frozen in liquid nitrogen, then after vortexing and separation by sieving heads and bodies were collected and pulverized in liquid nitrogen using a mortar and pestle. Once liquid nitrogen had evaporated the homogenate was transferred to a beaker containing 5 ml ice cold buffer A + NP (60 mM KCl, 15 mM NaCl, 15 mM HEPES pH7.6, 1 mM EDTA, 0.1 mM EGTA, with freshly added 0.15 mM spermine, 0.5 mM spermidine, 0.5 mM DTT, 0.5% NP-40 and 12 mM EDTA) and incubated on ice for 5 min with gentle stirring. The suspension was then transferred to a Dounce homogenizer (Wheaton) and disrupted with 20 strokes of a teflon pestle. The homogenate was filtered through 2 layers of Miracloth (Merck) into a 50 ml centrifuge tube. The pellet was scraped from the Miracloth, homogenized in 3 ml A + NP buffer and filtered through the same Miracloth. 2 ml buffer AS (60 mM KCl, 15 mM NaCl, 1 mM EDTA, 0.1 mM EGTA with freshly added 0.15 mM spermine, 0.5 mM spermidine, 0.5 mM DTT and 0.3 M saccharose) was added to the filtrate, which was then centrifuged at 4 °C , 3000 rpm for 5 min. The pellet was resuspended in 5 ml buffer A + NP, homogenized with 10 strokes in the Dounce homogenizer, then 2 ml AS buffer was added and the suspension was centrifuged at 4 °C, 3000 rpm for 5 min. The pellet was resuspended in 5 ml buffer A (60 mM KCl, 15 mM NaCl, 1 mM EDTA, 0.1 mM EGTA, 15 mM Tris–HCl pH7.4 with freshly added 0.15 mM spermine, 0.5 mM spermidine and 0.5 mM DTT), homogenized with 10 strokes in the Dounce homogenizer and centrifuged at 4 °C, 3000 rpm for 5 min. The pellet was resuspended in 500 µl buffer A + 0.1% NP (60 mM KCl, 15 mM HEPES pH7.6, 1 mM EDTA, 0.1 mM EGTA and 0.1% NP-40). 100 µl sample was set aside for concentration measurement. To crosslink chromatin 1 ml buffer A + 0.1% NP and 50 µl 30% formaldehyde was added to the remaining 400 µl homogenate and incubated at room temperature while shaking at 500 rpm for 10 min. To stop the reaction 150 µl 1 M glycine was added and the homogenate was centrifuged at 4 °C, 3000 rpm for 5 min. The pellet was resuspended in 5 ml buffer A + 0.1% NP then centrifuged at 4 °C, 3000 rpm for 5 min. The pellet was resuspended in 400 µl buffer A + 0.1% NP and stored at − 80 °C. To determine chromatin concentration the 100 µl sample that was set aside before crosslinking was centrifuged at 4 °C, 3000 rpm for 5 min, then the pellet was resuspended in 160 µl Nuclear Lysis Buffer (50 mM Tris–HCl pH 8.0, 1% SDS, 10 mM EDTA) and DNA concentration was measured using Qubit dsDNA HS Assay Kit (TFS).

### Micrococcal nuclease (MNase) digestion

Nuclei stored in buffer A + 0.1% NP were centrifuged at 3000 rpm for 5 min and resuspended in TE buffer. Nuclear suspension containing 1.5 µg DNA was digested with MNase (TFS) in a 20 µl enzyme reaction mix containing 0.02 U MNase at 37 °C for 30 min with shaking at 500 rpm. After addition of 10 mM EDTA and 100 µg/ml RNase A (TFS) samples were further incubated at 37 °C with shaking at 500 rpm for 30 min. Reverse crosslinking of samples was done at 65 °C with shaking at 500 rpm overnight, then 1 mg/ml Proteinase K (TFS) was added and samples were incubated at 37 °C with shaking at 500 rpm for 2 h.

### qPCR analysis

Primer pairs were designed to the 5′ HSEs^26^ and the coding sequence of the *Hsp27* gene (Supplementary Table S1). Digested chromatin was diluted 1:10 and used for qPCR by the SYBR green method with Luminaris Color HiGreen qPCR Master Mix (TFS) in a PikoReal Real-Time PCR System (TFS). qPCR data were normalized to the undigested samples. For statistical analysis of expression values Student’s t-test was performed.

## Supplementary Information

Supplementary Information.

## Data Availability

All relevant data are within the manuscript and its Supporting Information files. RNA-Seq data are deposited to the National Center for Biotechnology Information Sequence Read Archive (NCBI SRA) under accession PRJNA635790. Other datasets used and/or analyzed during the current study are available from the corresponding author on reasonable request.
