# Supplementary material for: The histone replacement gene His4r is involved in heat stress induced chromatin rearrangement
Source: Sci Rep. 2021 Mar 1;11:4878. doi: 10.1038/s41598-021-84413-4 (PMC7921661; doi:10.1038/s41598-021-84413-4)
Supplement: Supplementary file 1 — Supplementary Information. [file 41598_2021_84413_MOESM1_ESM.pdf]

The histone replacement gene *His4r* is involved in heat stress induced chromatin rearrangement

Anikó Faragó[1,2], Adél Ürmösi[1,2], Anita Farkas[1,2], László Bodai[1,*]

[1] Department of Biochemistry and Molecular Biology, Faculty of Science and Informatics, University of Szeged, 6726 Szeged Közép fasor 52. Hungary

[2] Doctoral School in Biology, Faculty of Science and Informatics, University of Szeged, 6726 Szeged, Hungary

* Corresponding author

E-mail: bodai@bio.u-szeged.hu

SUPPLEMENTARY INFORMATION

Supplementary Data S1. DNA sequence of wild-type *His4r*, *His4r*$^{\Delta 42}$ and *His4*^{rev5}

(A) wild type His4r gene region >3R:14625755..14626614

```
GTATTTTGAGGTGTGCGAGATCTGGTCACGCTCACAAGCAATTTTCTGGAGATTTCTTGAAGTGAGTTTTGTTGCTGAGAA
ATTTAAGTAAATAATAGAAGAATCGGTCAACAAAGTTTTTTTGTGCATAAACATAAAAAAAATAATTGGAGACCATTATTT
TGTATGAAAACAGACTTATTAACAACAGTTTTCACCTGTCAAATGAACGTTTACCTTTCACCAAACATGGCGTCGCTTTAC
AGTTTATTGCGTCTTCTCTTCTTTTCAGCCAACTTTACGAAGCAACTGAGAAATGACTGGTCGTGGAAAGGGAGGCAAAGG
ATTGGGAAAGGGGGGCGCCAAGCGTCATCGTAAGGTGCTTCGTGATAACATCCAGGGTATCACCAAGCCTGCTATTCGCCG
TTTGGCTCGTCGCGGCGGTGTTAAGCGTATCTCTGGCTTGATTTACGAGGAAACTCGCGGTGTGCTAAAGgtacgttctgg
cgaaacgaagggaaagtggtgaaaaactgattaatttctcggaaaattcacagGTATTCCTTGAGAACGTTATCCGTGACG
CTGTCACCTACACCGAGCACGCCAAGCGCAAGACCGTGACCGCCATGGACGTGGTCTATGCCCTCAAGCGCCAGGGACGCA
CCCTTTACGGATTTGGCGGTTAAGCACGCCCTCTAACCTCCACATAATAAATAACCATGGAGTTAACTTAACTACATTCAA
AATACTTGATCCAATTGAATCTTAATCAGTAGTATTCAACGCAAACTTAACCGAACGCAAATATTTTATAATTTCACAATA
TACGGACTAAATAAATCAAAAGATATCACATAGAGAACTCAATATCATGC
```

- UTR
- CDS
- intron

(B) His4r$^{\Delta 42}$

```
GTATTTTGAGGTGTGCGAGATCTGGTCACGCTCACAAGCAATTTTCTGGAGATTTCTTGAAGCATGATGAAATAACATTAS
TATTAGTATTTGAGTTTTGTTGCTGAGAAATTTAAGTAAATAATAGAAGAATCGGTCAACAAAGTTTTTTTGTGCATAAAC
ATAAAAAAAATAATTGGAGACCATTATTTTGTATGAAAACAGACTTATTAACAACAGTTTTCACCTGTCAAATGAACGTTT
ACCTTTCACCAAACATGGCGTCGCTTTACAGTTTATTGCGTCTTCTCTTCTTTTCAGCCAACTTTACGAAGCAACTGAGAA
ATGACTGGTCGTGGAAAGGGAGGCAAAGGATTGGGAAAGGGGGGCGCCAAGCGTCATCGTAAGGTGCTTCGTGATAACATC
CAGGGTATCACCAAGCCTGCTATTCGCCGTTTGGCTCGTCGCGGCGGTGTTAAGCGTATCTCTGGCTTGATTTACGAGGAA
ACTCGCGGTGTGCTAAAGgtacgttctggcgaaacgaagggaaagtggtgaaaaactgattaatttctcggaaaattcaca
gGTATTCCTTGAGAACGTTATCCGTGACGCTGTCACCTACACCGAGCACGCCAAGCGCAAGACCGTGACCGCCATGGACGT
GGTCTATGCCCTCAAGCGCCAGGGACGCACCCTTTACGGATTTGGCGGTTAAGCACGCCCTCTAACCTCCACATAATAAAT
AACCATGGAGTTAACTTAACTACATTCAAAATACTTGATCCAATTGAATCTTAATCAGTAGTATTCAACGCAAACTTAACC
GAACGCAAATATTTTATAATTTCACAATATACGGACTAAATAAATCAAAAGATATCACATAGAGAACTCAATATCATGC
```

- wild type His4r gene region
- **29 bp P ELEMENT DERIVED SEQUENCE**
- 694 bp DELETED SEQUENCE

(C) His4r^{rev5}

GTATTTTGAGGTGTGCGAGATCTGGTCACGCTCACAAGCAATTTTCTGGAGATT**TCTTGAAG**CATGATGAAATAACAT*AAC*
*ATAA*CATGTTATTTCATCATG**TCTTGAAG**TGAGTTTTGTTGCTGAGAAATTTAAGTAAATAATAGAAGAATCGGTCAACAA
AGTTTTTTTGTGCATAAACATAAAAAAAATAATTGGAGACCATTATTTTGTATGAAAACAGACTTATTAACAACAGTTTTC
ACCTGTCAAATGAACGTTTACCTTTCACCAAACATGGCGTCGCTTTACAGTTTATTGCGTCTTCTCTTCTTTTCAGCCAAC
TTTACGAAGCAACTGAGAAATGACTGGTCGTGGAAAGGGAGGCAAAGGATTGGGAAAGGGGGGCGCCAAGCGTCATCGTAA
GGTGCTTCGTGATAACATCCAGGGTATCACCAAGCCTGCTATTCGCCGTTTGGCTCGTCGCGGCGGTGTTAAGCGTATCTC
TGGCTTGATTTACGAGGAAACTCGCGGTGTGCTAAAGgtacgttctggcgaaacgaagggaaagtggtgaaaaactgatta
atttctcggaaaattcacagGTATTCCTTGAGAACGTTATCCGTGACGCTGTCACCTACACCGAGCACGCCAAGCGCAAGA
CCGTGACCGCCATGGACGTGGTCTATGCCCTCAAGCGCCAGGGACGCACCCTTTACGGATTTGGCGGTTAAGCACGCCCTC
TAACCTCCACATAATAAATAACCATGGAGTTAACTTAACTACATTCAAAATACTTGATCCAATTGAATCTTAATCAGTAGT
ATTCAACGCAAACTTAACCGAACGCAAATATTTTATAATTTCACAATATACGGACTAAATAAATCAAAAGATATCACATAG
AGAACTCAATATCATGC

- **8 bp TARGET SITE DUPLICATION**
- **40 bp P ELEMENT DERIVED SEQUENCE**

Supplementary Figure S1. Original photo of the full-length gel presented on Fig. 1B.

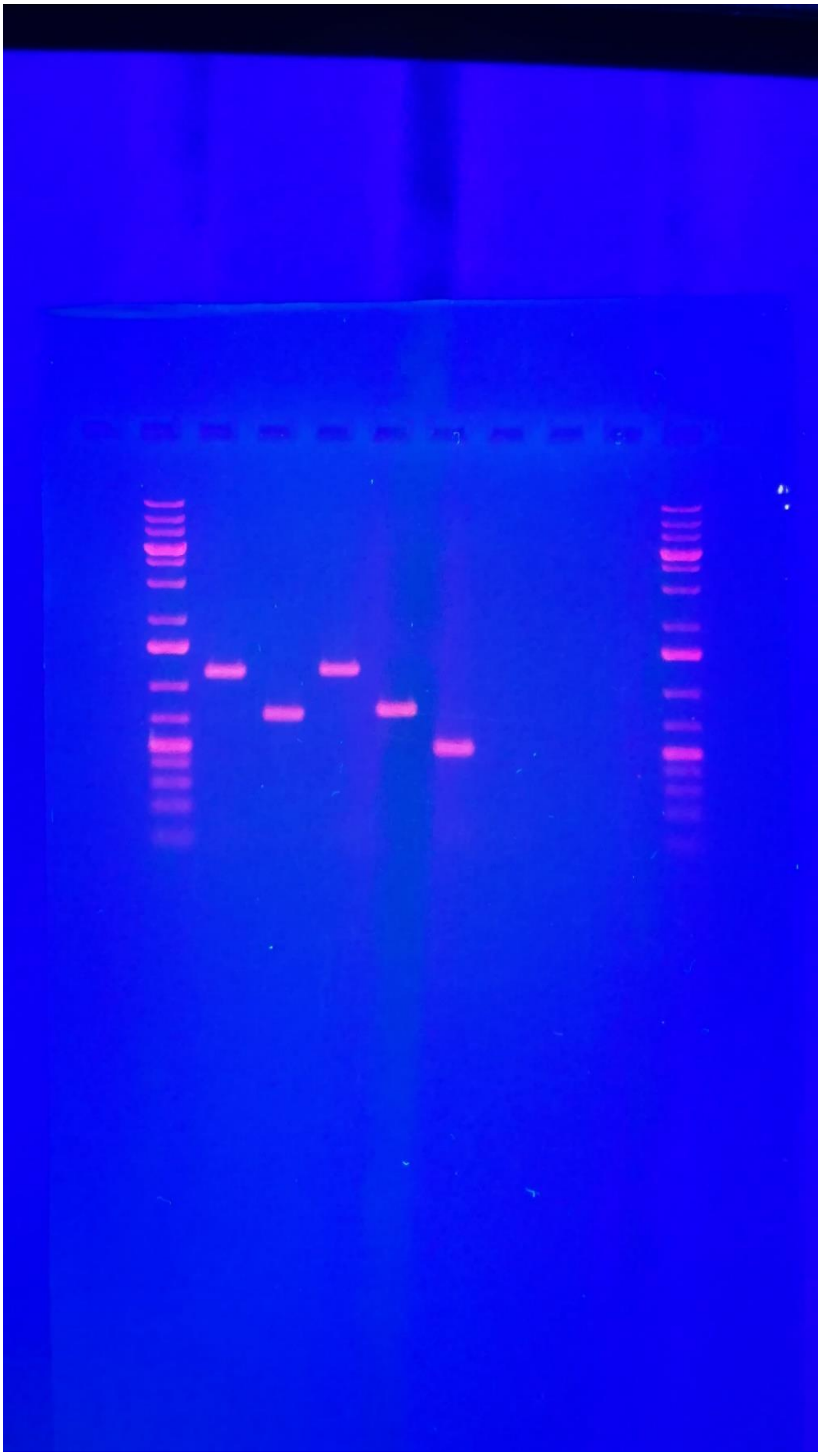

Supplementary Figure S2. Complementation test. Both male or female *w; His4r*^{LL05512}/*His4r*$^{\Delta 42}$ heterozygotes crossed with *w*^{1118} flies resulted in viable progeny rebutting former finding [20] that loss of *His4r* causes sterility.

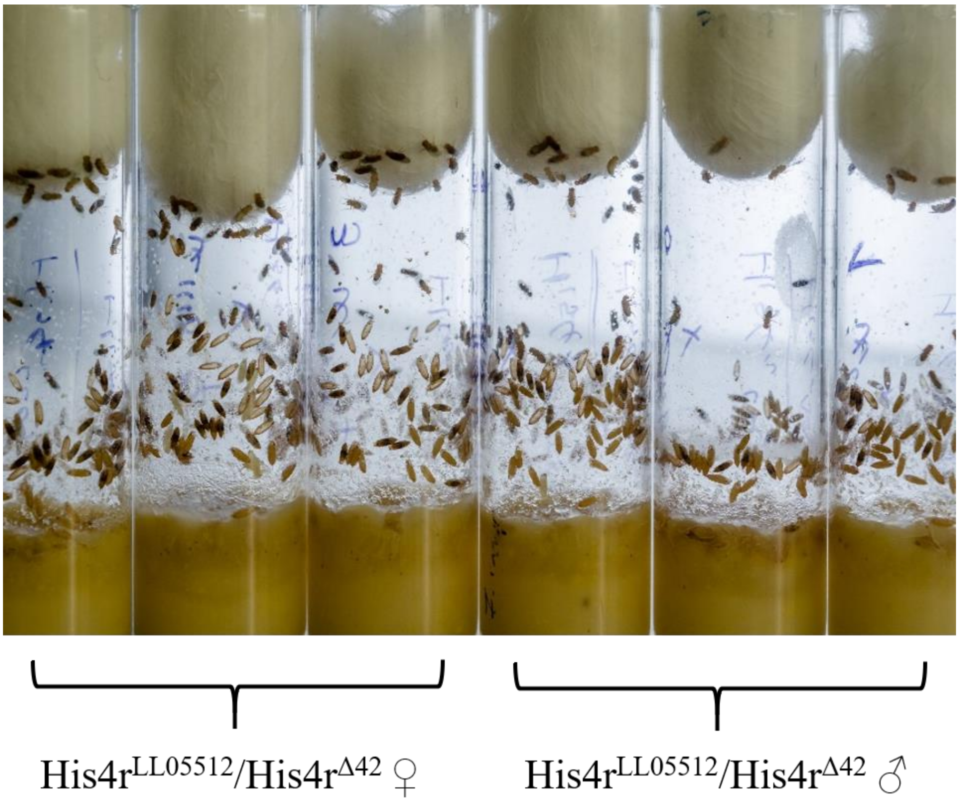

Supplementary Figure S3. GO Biological Process term classification of genes up-regulated in *His4r*$^{\Delta 42}$ mutants. The graph shows the number of misregulated genes belonging to PANTHER GO-Slim categories based on GO Ontology database (2019-10-08 release).

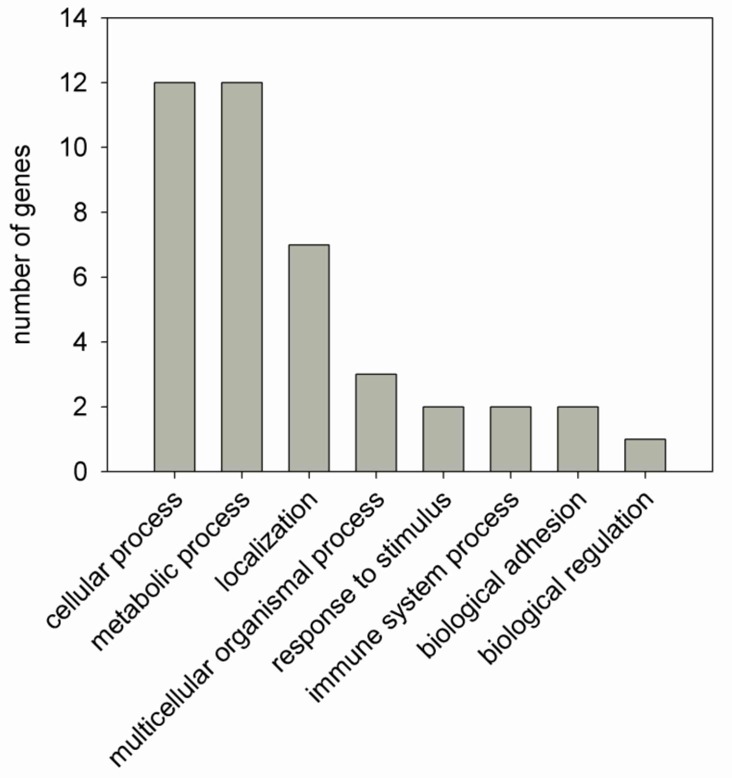

Supplementary Figure S4. GO Biological Process term classification of genes down-regulated in *His4r*$^{\Delta 42}$ mutants. The graph shows the number of misregulated genes belonging to PANTHER GO-Slim categories based on GO Ontology database (2019-10-08 release).

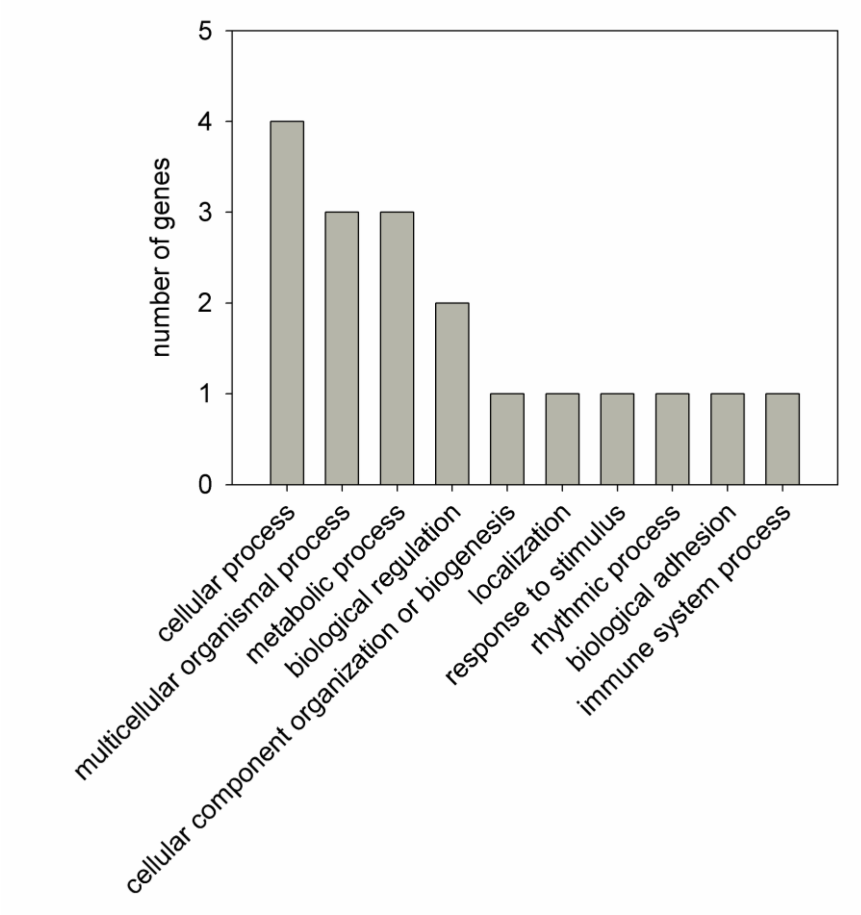

Supplementary Figure S5. GO Molecular Function term classification of genes up-regulated in *His4r*$^{\Delta42}$ mutants. The graph shows the number of misregulated genes belonging to PANTHER GO-Slim categories based on GO Ontology database (2019-10-08 release).

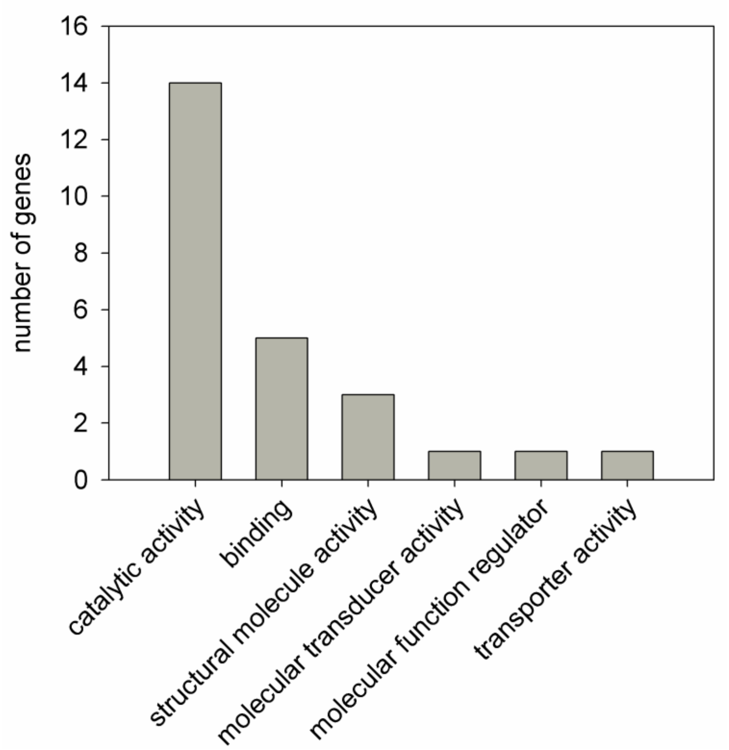

Supplementary Figure S6. GO Molecular Function term classification of genes down-regulated in *His4r*$^{\Delta 42}$ mutants. The graph shows the number of misregulated genes belonging to PANTHER GO-Slim categories based on GO Ontology database (2019-10-08 release).

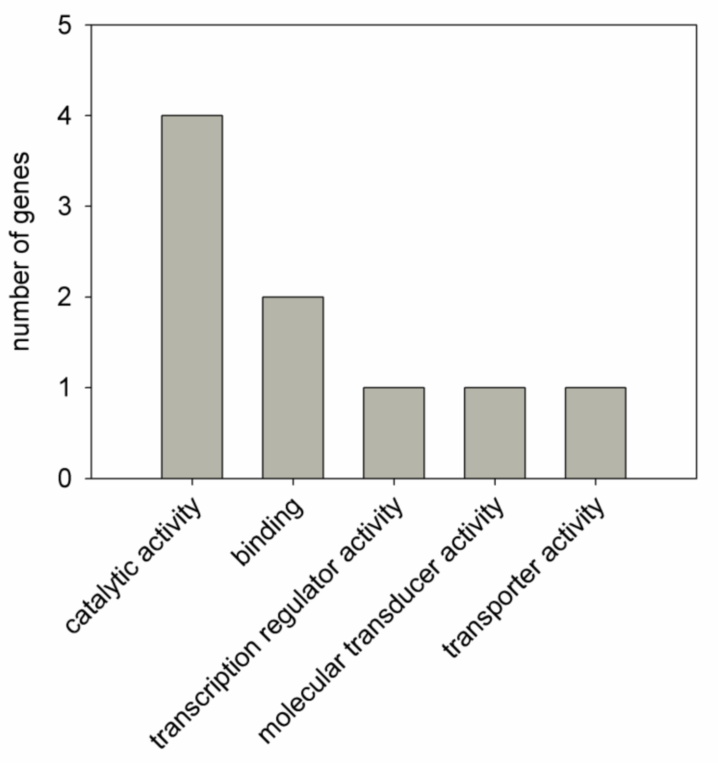

Supplementary Table S1. PCR primer sequences.

primer name	*primer 5'-3' sequence*
His4rFseq	TGG CCG ATA GGC GAT AAC TG
His4rgR	GGT ACC GGG AGA GCT AAA TTT GCA G
His4rE3CR	GAA TTC GCA CCG CCA AAT CCG TA
tubulin qF	TGT CGC GTG TGA AAC ACT TC
tubulin qR	AGC AGG CGT TTC CAA TCT G
His4r qF	TCG CGG TGT GCT AAA GGT ATT C
His4r qR	AAT CCG TAA AGG GTG CGT CC
Hsp27 qF	GCT TTG ACC CCA ACG AGG TA
Hsp27 qR	CCA TTT GAG CGT CAA GGC AC
Hsp60A qF	TTT GCC AGT TTC GCT TGC TC
Hsp60A qR	CCC CAC GAC TGC TCA ATG AT
Hsp68 qF	AAG TGT TTG AGG GCG AGA GG
Hsp68 qR	TCA GGA TAC CGT TTG CGT CC
Hsp83 qF	AGG TGA TCC GCA AGA ACC TG
Hsp83 qR	GAG GTG TGG AAG CGA AGG AA
Hsp27-HSE qF	ACT CCC CAG AAA AGA AAT GTC AAG A
Hsp27-HSE qR	CTC TCG CAT CTT CTG GCT CTT T
